# Evidence for Widespread Class II Microcins in *Enterobacterales* Genomes

**DOI:** 10.1128/aem.01486-22

**Published:** 2022-11-17

**Authors:** T. Jeffrey Cole, Jennifer K. Parker, Aaron L. Feller, Claus O. Wilke, Bryan W. Davies

**Affiliations:** a Department of Integrative Biology, The University of Texas at Austin, Austin, Texas, USA; b Department of Molecular Biosciences, The University of Texas at Austin, Austin, Texas, USA; c John Ring LaMontagne Center for Infectious Diseases, The University of Texas at Austin, Austin, Texas, USA; University of Michigan—Ann Arbor

**Keywords:** *Enterobacteriaceae*, bacteriocins, bioinformatics, computational biology, Gram-negative bacteria, microcin

## Abstract

Microcins are a class of antimicrobial peptides produced by certain Gram-negative bacterial species to kill or inhibit the growth of competing bacteria. Only 10 unique, experimentally validated class II microcins have been identified, and the majority of these come from Escherichia coli. Although the current representation of microcins is sparse, they exhibit a diverse array of molecular functionalities, uptake mechanisms, and target specificities. This broad diversity from such a small representation suggests that microcins may have untapped potential for bioprospecting peptide antibiotics from genomic data sets. We used a systematic bioinformatics approach to search for verified and novel class II microcins in E. coli and other species within its family, *Enterobacteriaceae*. Nearly one-quarter of the E. coli genome assemblies contained one or more microcins, where the prevalence of hits to specific microcins varied by isolate phylogroup. E. coli isolates from human extraintestinal and poultry meat sources were enriched for microcins, while those from freshwater were depleted. Putative microcins were found in various abundances across all five distinct phylogenetic lineages of *Enterobacteriaceae*, with a particularly high prevalence in the “Klebsiella” clade. Representative genome assemblies from species across the *Enterobacterales* order, as well as a few outgroup species, also contained putative microcin sequences. This study suggests that microcins have a complicated evolutionary history, spanning far beyond our limited knowledge of the currently validated microcins. Efforts to functionally characterize these newly identified microcins have great potential to open a new field of peptide antibiotics and microbiome modulators and elucidate the ways in which bacteria compete with each other.

**IMPORTANCE** Class II microcins are small bacteriocins produced by strains of Gram-negative bacteria in the *Enterobacteriaceae*. They are generally understood to play a role in interbacterial competition, although direct evidence of this is limited, and they could prove informative in developing new peptide antibiotics. However, few examples of verified class II microcins exist, and novel microcins are difficult to identify due to their sequence diversity, making it complicated to study them as a group. Here, we overcome this limitation by developing a bioinformatics pipeline to detect microcins *in silico*. Using this pipeline, we demonstrate that both verified and novel class II microcins are widespread within and outside the *Enterobacteriaceae*, which has not been systematically shown previously. The observed prevalence of class II microcins suggests that they are ecologically important, and the elucidation of novel microcins provides a resource that can be used to expand our knowledge of the structure and function of microcins as antibacterials.

## INTRODUCTION

Virtually all natural environments contain bacterial communities where diverse species compete with their neighbors for space and resources. Among the various strategies for interacting and interfering with one another (1), the production of bacteriocins is particularly widespread (2). Bacteriocins are a large and functionally diverse group of proteinaceous and peptidic toxins that are typically inhibitory toward close relatives (3). They have been studied extensively among Gram-positive species (4) and have served as a source of inspiration for developing antibiotics and preservatives (5–8). Gram-negative bacteriocins have been studied far less, and knowledge is especially limited about the Gram-negative peptidic group known as microcins (9, 10).

Microcins (11) are small (<10-kDa) bacteriocins known to be produced by the *Enterobacteriaceae*. They are divided into two classes: the smaller, heavily posttranslationally modified class I microcins (12) and the larger class II microcins, which are unmodified except for disulfide bonds (class IIa) (13) or minimally modified (class IIb) (14). Because of their simplified structure and common production and export machinery, class II microcins are easier to manipulate and can be secreted heterologously (15–17). They are generally understood to play a role in interbacterial competition (18–20), are capable of modulating bacterial communities *in vivo* (21, 22), and may be useful in the development of antibacterials to target Gram-negative pathogens (10). Despite this, there are only 10 class II microcins with confirmed antibacterial activity and partial characterization, limiting our understanding of their functions and potential utility. Of these, eight were identified in Escherichia coli, and two were identified in Klebsiella pneumoniae. Most of the source isolates were acquired from feces (23, 24) or are fecally associated (e.g., uropathogenic bacteria) (25), and microcin screening was conducted primarily via visual observation using the traditional culture-based zone-of-inhibition method (23, 24). These methods of discovery inherently bias the types of microcins observed and limit the screening throughput.

The production and export machinery of class II microcins consists of five genetic components (26–28): (i) the precursor peptide, containing an N-terminal secretion signal sequence fused to the core microcin structural component; (ii) a cognate immunity protein to provide immunity to the producing cell; (iii) a C39 peptidase-containing ABC transporter (PCAT); (iv) a membrane fusion protein (MFP); and (v) TolC, an outer membrane efflux protein. The PCAT recognizes and cleaves the microcin signal sequence and provides a connection to the MFP, which connects to TolC to complete export beyond the cell membrane (28, 29). Although the amino acid sequences of microcin core peptides and immunity proteins are highly variable, the sequences of PCATs and MFPs are highly conserved. The signal sequences of the precursor peptides also show some conservation and are characterized by a terminal “double-glycine” (can be either GG or GA) cleavage site (13).

It seems unlikely that class II microcins exist only in E. coli and Klebsiella pneumoniae; as these species belong to sister genera, it suggests that microcins may exist in other closely related taxa. Furthermore, the PCAT needed for export is widely distributed among Gram-negative bacteria (30). The conservation of the microcin export components suggests that it is possible to perform a sequence-based search for verified and novel microcins among whole-genome sequence data, a method unavailable when microcins were initially discovered in the 1980s and 1990s. Previous efforts to bioinformatically predict class II microcins or other similar Gram-negative peptides are limited. A single study screened 79 Gram-negative genomes for peptides with a double-glycine signal sequence motif in proximity to an ABC transporter with a C39 peptidase domain (31). However, that study, completed in 2003, used a training data set composed mostly of functionally unconfirmed signal sequences from proteins of unknown function due to the lack of available data. At that point, only five class II microcins had been identified, three of which were incorporated into the training data set (32). Since that study, the number of confirmed microcins, knowledge of microcin characteristics, and the number of available genome assemblies for screening have increased such that a specific and more robust search, focused on class II microcins, can be implemented.

Here, we developed a rapid, high-throughput bioinformatics pipeline for the identification of putative class II microcins and their associated molecular machinery among bacterial genome assemblies. The goals were 2-fold: (i) to provide a means to identify verified and novel microcins and (ii) to examine the extent to which microcins are found in different species, phylogroups, and habitats, both within and outside the family *Enterobacteriaceae*. With our pipeline, we sought to overcome the inadequacies that arise from using general bacteriocin detection software for the purpose of detecting microcins by incorporating up-to-date knowledge of microcin sequence characteristics using a hidden Markov model (HMM)-based approach. We began by investigating E. coli strains spread across different phylogenetic lineages and habitats to determine how microcins are distributed within this species and if they have a higher prevalence in certain lineages or habitats. We continued our investigation moving progressively outward and interrogated genomes throughout the *Enterobacteriaceae*, *Enterobacterales*, *Vibrionaceae*, and *Pasteurellaceae*. We found that microcins are widely dispersed both within the family *Enterobacteriaceae* and outside it, demonstrating that microcin production is not restricted to enterobacterial species and gut environments. This suggests that microcins may play a role in competition among numerous species in different microbial communities.

## RESULTS

### Microcin identification pipeline.

**FIG 1:**
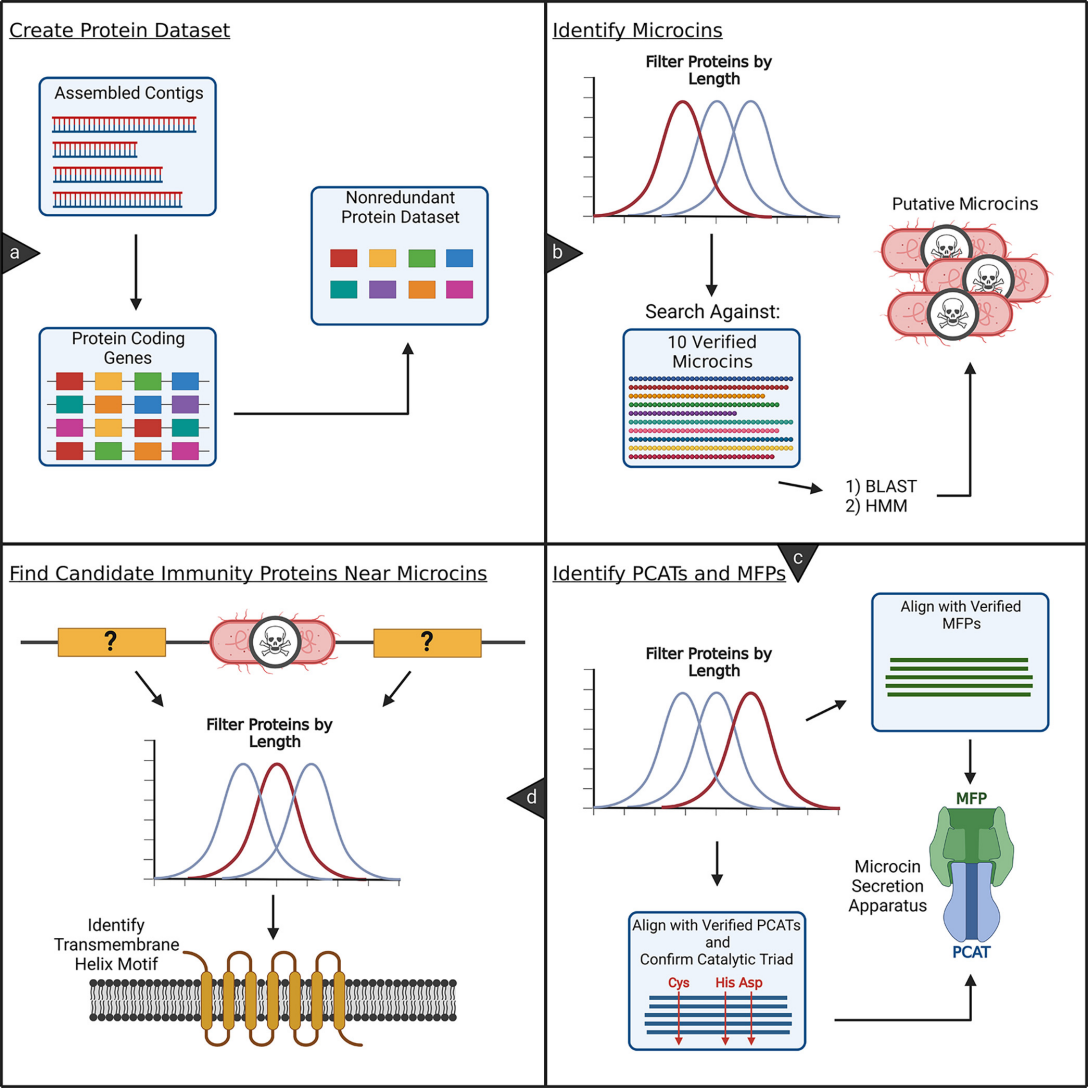
Bioinformatics workflow diagram. Each panel contains an overview of one of the four main modules of the cinful bioinformatics pipeline. (a) Assembled genomic contigs are provided as input. Prodigal is used to generate protein-coding gene predictions. Per sample, a nonredundant set of protein sequences is generated. (b) Protein-coding genes are filtered by a predefined length. Microcin homologs are then identified via a homology search against a verified set of microcin sequences using BLAST followed by HMMER. (c) To identify putative PCAT and MFP homologs, the input sequences are filtered by a preset length criterion. Next, a homology search is performed using DIAMOND. Homologs are further inspected for complete homology by comparing them to a multiple-sequence alignment of verified PCATs or MFPs, retaining only hits that match the rest of the alignment by a defined threshold. PCATs are further screened by matching residues at three conserved positions that function as a catalytic triad. (d) Candidate immunity proteins are identified by proximity in the genome to putative microcins and a predefined length criterion. The detection of a transmembrane motif is provided as supporting information but is not a screening criterion. (Figure created with BioRender.com.)

**FIG 2:**
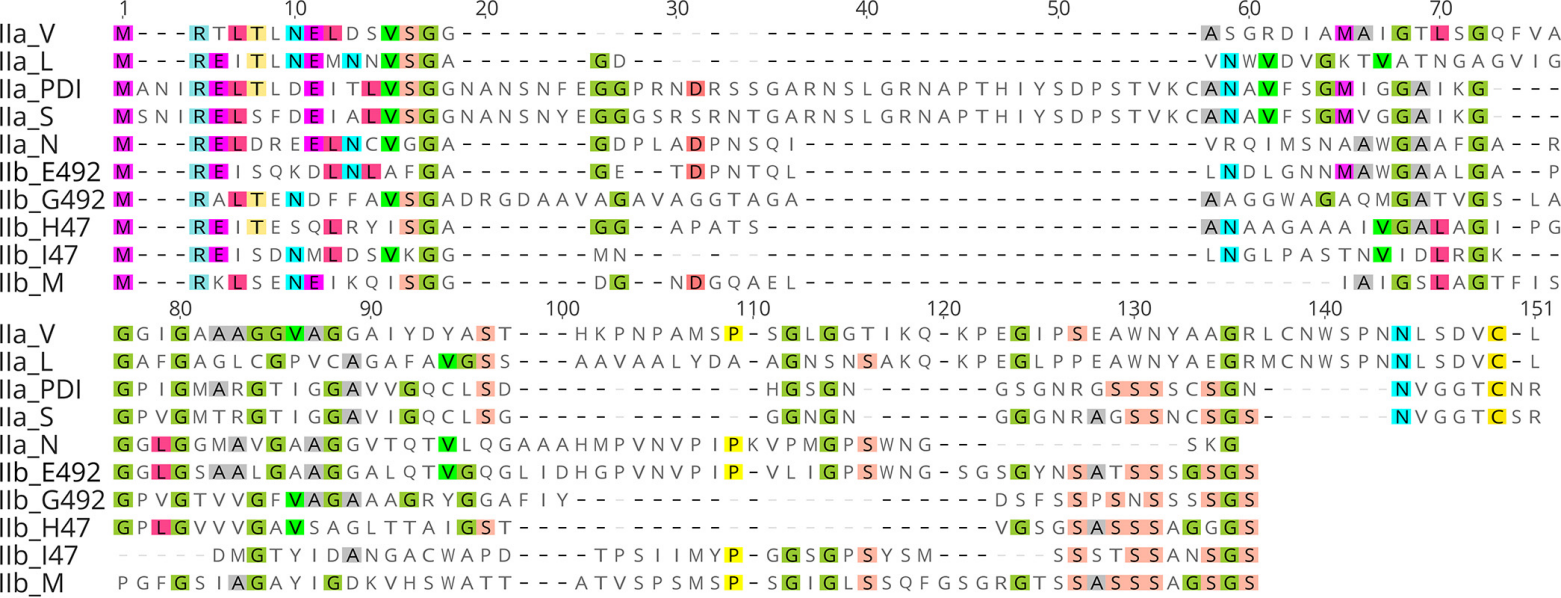
Class II microcin multiple-sequence alignment (MSA). An MSA of the 10 verified class II microcin sequences was constructed with MAFFT. Residues matching the majority consensus are highlighted. Microcin assignment to class IIa versus class IIb is indicated in the sequence name; class IIb microcins are differentiated by their glycine- and serine-rich C-terminal region, which is evident in this alignment.

We developed a bioinformatics pipeline to identify putative class II microcins and their associated machinery (PCAT, MFP, and candidate immunity proteins) in bacterial genome assemblies (Fig. 1). We refer to the pipeline that we have developed as cinful, a portmanteau of micro*cin ful*l. A primary component of the pipeline that we developed involves the 10 verified class II microcin sequences. The multiple-sequence alignment (MSA) for these proteins is visualized in Fig. 2. This MSA was used to generate a profile hidden Markov model (pHMM) with HMMER to identify other class II microcins.

### Survey of microcins in the E. coli pangenome.

**FIG 3:**
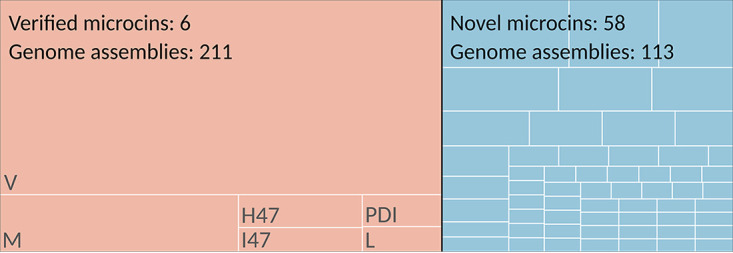
E. coli assemblies with microcin hits. An overview of the distribution of E. coli genome assemblies that contain verified or novel microcins is shown. The relative sizes of the two boxes outlined in black represent the relative numbers of genome assemblies that had verified microcin hits identical to the 10 that have already been functionally characterized (left) or novel microcin hits with sequences that are different from but similar to those that have been verified (right). The relative sizes of the boxes outlined in white within these two boxes are proportional to the numbers of hits identified per unique sequence. For the verified microcins, their identity is indicated in gray text in the appropriate box.

Of the microcins that have been functionally characterized, 8 of 10 were originally found in E. coli. The extent to which microcins persist throughout this species has not been characterized in depth. An ideal data set to explore microcins in E. coli is from a recent phylogenomic investigation that includes genome assemblies from E. coli isolates representing several independently evolving lineages (phylogroups) across different host-associated and freshwater sources in Australia (33). Genome assemblies from 1,224 E. coli isolates were provided as the input to our bioinformatics pipeline. A total of 288 (23.5%) of the assemblies contained one or more microcin hits. Of these 288 assemblies, 211 contained sequences identical to those of verified microcins, while 113 contained novel homologous putative microcins (36 assemblies contained both verified and novel microcins) (Fig. 3). The total number of microcins identified from these assemblies was 365, with 220 verified hits (representing 6 verified microcin sequences) and 145 novel hits (representing 58 unique sequences). The number of hits per assembly ranged from 1 to 5, and 56 (19.4%) of the microcin-containing assemblies had multiple microcin hits. Given that cinful outputs the best microcin hit per contig, this could be an undercount if multiple microcins are present on the same contig. These data are available in Supplemental File 1.

Seven of the eight microcins of E. coli origin were found among these E. coli assemblies. Including both verified and novel hits, those closest to microcin V occurred the most frequently (57.8% of hits), followed by microcins M (15.6%), H47 (10.7%), PDI (6.8%), I47 (6.3%), L (1.9%), and S (0.5%). When accounting for only hits to verified microcins, microcin V had an even higher prevalence (77.3%). No hits to microcin N were found. For the two microcins of K. pneumoniae origin (E492 and G492), a single hit to microcin E492 was identified. The scarcity of these microcins suggests that they and their variants are not usually present among E. coli isolates and may be more commonly carried by K. pneumoniae.

Most novel microcins identified were relatively similar in sequence (>90% pairwise identity) to the verified microcin by which they were identified. This is not surprising given the very small training data set (*n* = 10); an HMM with a larger training set would likely identify more diverse hits. Among the 58 unique novel microcin sequences, 18 (31.0%) have 100% identity in the BLAST hit region but are nonidentical to the verified microcin (i.e., sequences that are fragments of the verified microcin or contain additional amino acids, some of which appear to be caused by incorrect start codon identification, resulting in a short insertion at the N terminus of the microcin). Excluding these 18 sequences, the average percent identity of novel microcins to their respective verified microcins within the hit region was 89.9%. Only 3/40 (7.5%) of these novel hits had <40% identity within the hit region to their verified microcin hit. A threshold of >40% identity is an often-suggested minimum for possible homology (34). An MSA of these 40 novel microcins with their respective verified microcins to which they were a hit is available in Supplemental File 2A.

**FIG 4:**
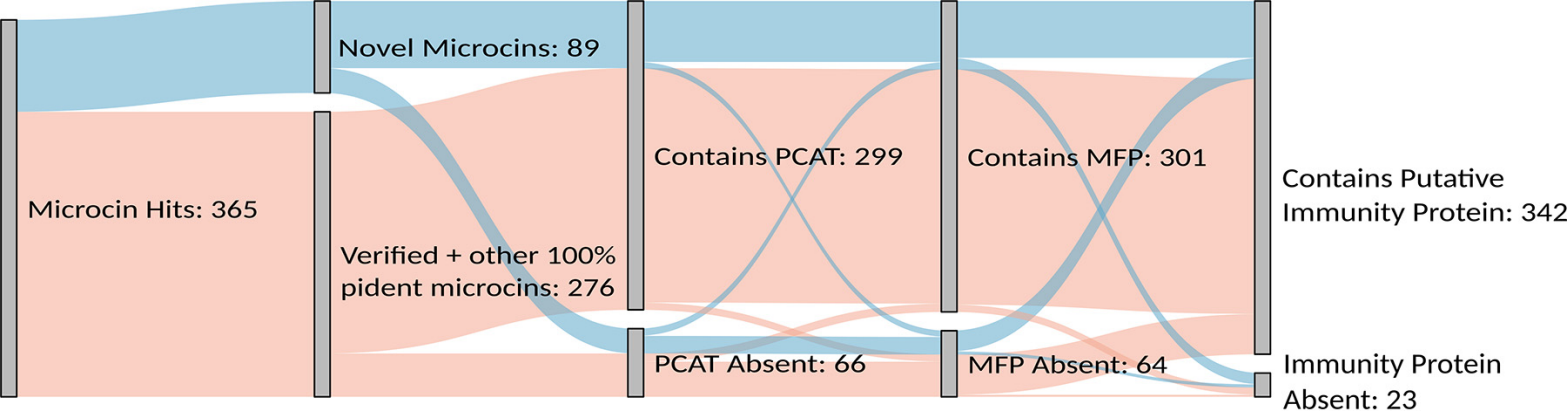
Microcin hits from E. coli assemblies and their associated export machinery and candidate immunity proteins. A Sankey diagram of the verified and novel microcin hits from E. coli assemblies, with further filters that include whether the microcin had a PCAT within the same assembly, an MFP within the same assembly, and/or an immunity protein candidate on the same contig as the microcin, is shown. Included with the verified microcin hits are other microcin hits having 100% identity (pident) to verified microcins but differing in length, as these are unlikely to be novel microcins. The bars are colored to indicate assemblies, with verified microcin sequences in pink and novel microcin sequences in blue.

Each microcin hit (*n* = 365) was further filtered based on the following criteria: (i) it had a 100% identity match to a verified microcin, (ii) it contained a PCAT homolog within the source assembly, (iii) it contained an MFP homolog within the source assembly, and (iv) it contained an immunity protein candidate on the same contig as that of the microcin hit. In this case, filtering microcins based on the percent identity match to a verified microcin was done so that the sequences that had 100% identity in the BLAST hit region but were nonidentical to the verified microcin (as discussed above) were grouped with verified microcins. This allowed the inclusion of all exact sequence matches without length requirements, meaning that shorter or longer hits with a 100% identity match were included with the verified microcins, as we do not consider these to be true novel microcins. This resulted in a mixture of assemblies that met varying numbers of these criteria, as illustrated in Fig. 4. For 81.9% of the microcin hits, a PCAT was identified within the source assembly. MFPs were identified at a similar rate (82.5% of hits). The detection of PCATs and MFPs supports that these putative microcins might be actively produced, as a microcin cannot be exported without these components. The large majority of the assemblies containing a PCAT also contained an MFP. The identification of at least one immunity protein candidate was successful for 93.7% of the microcin hits, with the caveat that this is the least restrictive of the searches for microcin-associated proteins due to the difficulty in identifying immunity proteins by homology.

**FIG 5:**
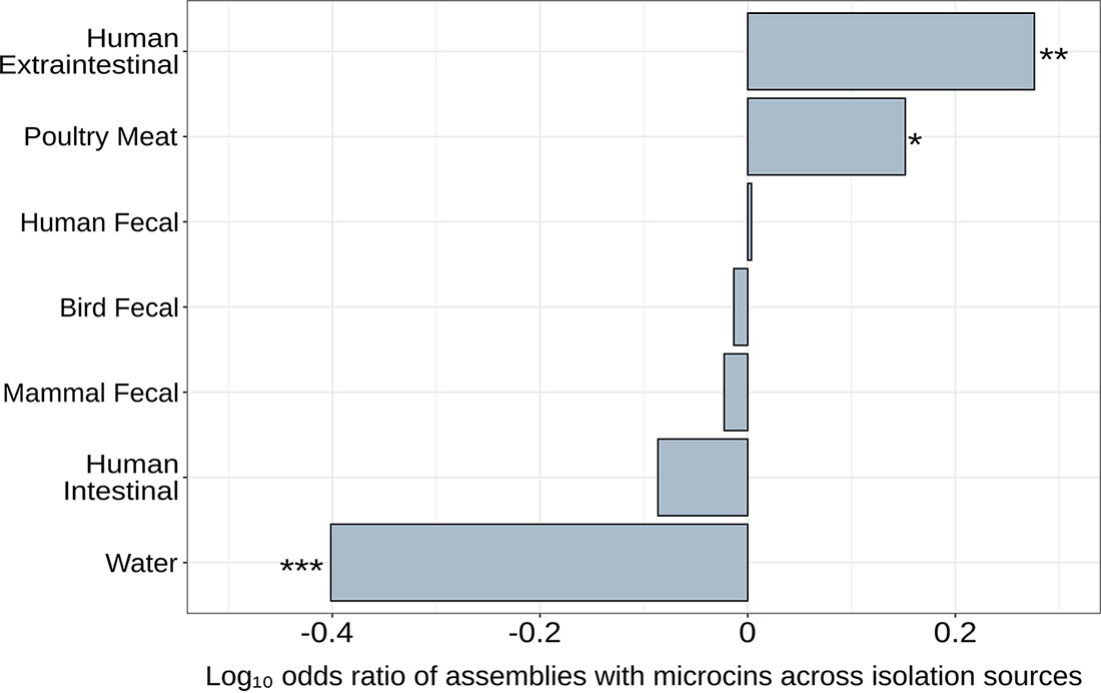
Enrichment of E. coli genome assembly strain categories for microcins. Strain categories represent the habitats from which the isolates with genome assemblies originated. Enrichment is calculated using the log_10_ of the proportion of assemblies of a given strain category with microcin hits relative to the global expected proportion of assemblies with microcin hits. Asterisks indicate the level of statistical significance (*, *P* < 0.05; **, *P* < 0.01; ***, *P* < 0.001).

E. coli assemblies were analyzed for the presence of microcins in relation to the habitat of the isolate. Of the 1,224 E. coli isolates with genome assemblies, 1,213 were known to originate from one of the following habitat sources: human extraintestinal (blood or urine), human intestinal (biopsy specimen), human fecal, nonhuman mammal fecal, bird meat, bird fecal, or freshwater (33). Analysis showed that 284 isolates from the selected habitat sources contained at least one microcin hit. A one-way chi-square test of the total number of assemblies compared to an equal frequency of assemblies per strain category revealed that the different environments were not equally represented in the genome assemblies (*c*^2^ = 241.17; df = 6; *P* < 2.2 × 10^−16^). A two-way chi-square test of the number of assemblies per environment relative to the number of microcins per environment shows the enrichment or depletion of microcins across isolation sources (*c*^2^ = 56.16; df = 6; *P* < 2.7 × 10^−10^). To demonstrate which habitats contain assemblies that are enriched for microcins, the relative number of microcins per habitat was compared to the expected percentage (284/1,213 = 23.4%) of microcins and then log transformed to give the log of the odds of a strain category containing assemblies with microcins. To quantify specifically which strain categories were enriched/depleted for microcins, pairwise Fisher’s exact tests were performed on the number of microcin hits and the number of genome assemblies with false discovery rate correction. E. coli isolates from freshwater were significantly depleted for microcins compared to all host-associated habitats, based on the expected value from an equal distribution (*P* = 1.98 × 10^−5^) (Fig. 5). Human extraintestinal sources were the most enriched for microcins (*P* = 4.28 × 10^−3^), and isolates from poultry meat were also significantly enriched but to a lesser extent (*P* = 2.77 × 10^−2^). All fecal/intestinal habitat categories had roughly average expected amounts of microcins and were neither enriched nor depleted.

**FIG 6:**
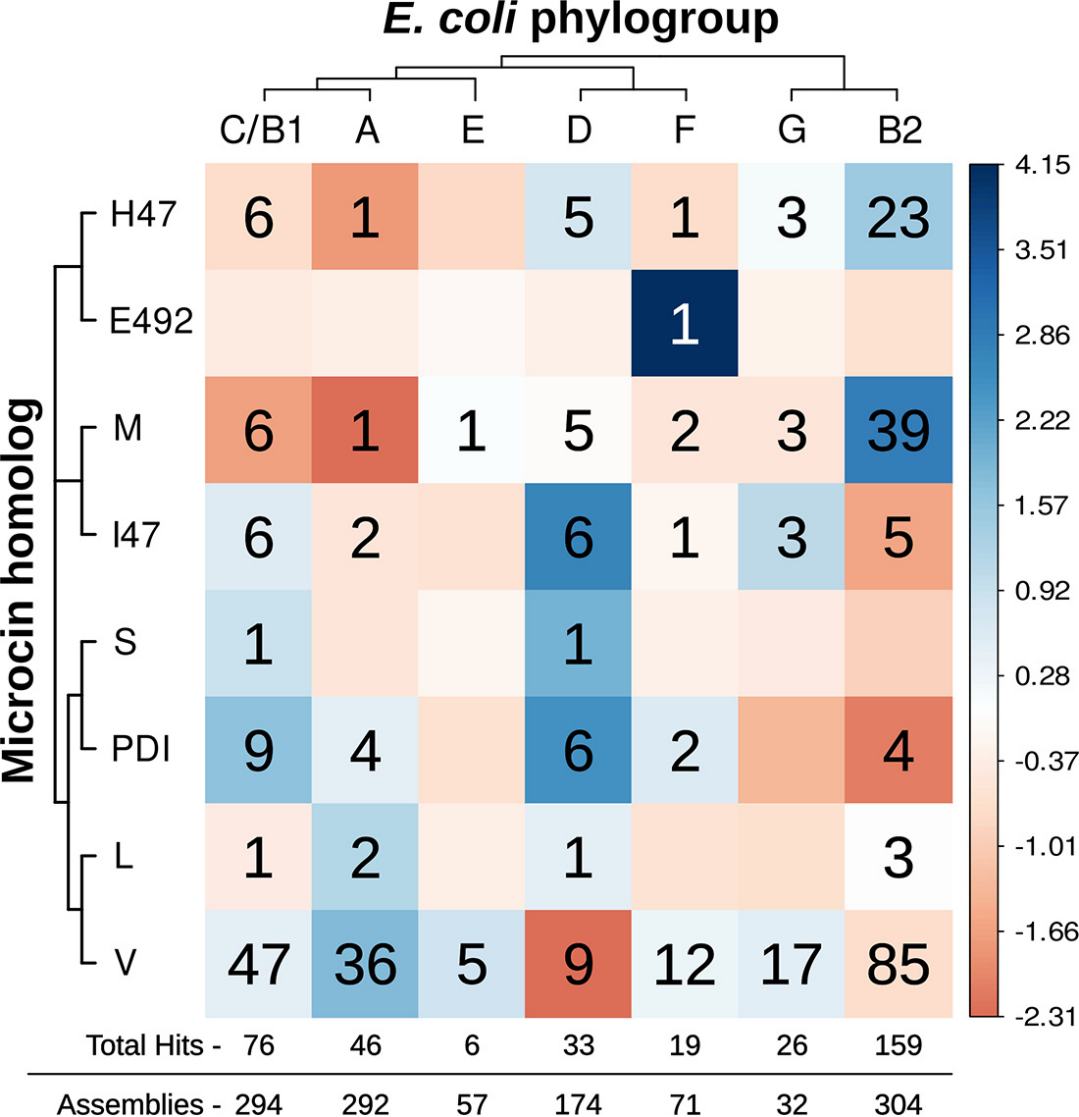
Distribution of hits to the 10 verified microcins across E. coli phylogroups. A count matrix of the number of microcin hits separated by the E. coli phylogroup and type of microcin homolog is shown. Phylogroups B1 and C were combined based on precedent from the original phylogenomic study of these isolates (33). Cladograms for the microcins and phylogroups represent phylogenetic relationships generated in this work and the work of Abram et al. (59), respectively. No hits were observed for microcins N and G492. Colors correspond to Pearson residuals in a global chi-square test, the difference between the observed and expected values divided by the square root of the expected value. Blue indicates enrichment and red indicates depletion compared to the expected values. The total hits identified and assemblies analyzed are represented below the count matrix.

Phylogroup assignment was also available for this E. coli data set (33). For the analysis of microcin prevalence by E. coli phylogroup, all 1,224 E. coli isolates were used, providing representation of all eight phylogroups (A, B1, B2, C, D, E, F, and G). The number of E. coli assemblies was not evenly distributed among the phylogroups (*c*^2^ = 700.2; df = 7; *P* < 2.2 × 10^−16^). The number of assemblies containing putative microcins (*n* = 288; 23.5%) was significantly different from the number that would be expected based on the relative frequencies of assemblies available per phylogroup (*c*^2^ = 118.67; df = 7; *P* < 2.2 × 10^−16^). Hits to 8 of the 10 verified microcins are shown by phylogroup (no hits to microcins N and G492 were found) (Fig. 6). Hits from phylogroups B1 and C were combined here, as was done in the original analysis of this data set (33). The specific microcin homolog matches varied between phylogroups. The only microcin homolog identified in all phylogroups was microcin V; it was the most enriched in phylogroup A and the most depleted in phylogroup D. Other phylogroups where specific microcins were particularly enriched were B2 (microcins M and H47) and D (microcins I47 and PDI). No phylogroup contained hits to all eight microcins, although B1/C and D did have seven out of eight microcins represented. Phylogroup B2 contained the highest total number of microcin hits (43.6% of hits), followed by phylogroup B1 (16.7%). However, when accounting for the different numbers of assemblies analyzed per phylogroup, the proportion of microcin hits relative to the number of assemblies analyzed was the highest for phylogroup G (0.81), followed by B2 (0.52). Interestingly, if phylogroups B1 and C are instead treated separately, phylogroup C has the highest proportion of microcin hits per assembly (0.94), versus when B1 and C are combined (0.26).

### Survey of microcins in *Enterobacteriaceae* genomes.

While 8 of the 10 verified class II microcins are from E. coli, 2 have been identified in Klebsiella pneumoniae, which belongs to a sister clade within the *Enterobacteriaceae*. To determine the extent to which microcins exist in species besides E. coli within this family, genome assemblies from species included in a recent phylogenomic investigation of *Enterobacteriaceae* were provided as the input to our bioinformatics pipeline to identify putative microcins (35). In that study, species within the *Enterobacteriaceae* were assigned to the following six clades: “Escherichia,” “Klebsiella,” “Enterobacter,” “*Kosakonia*,” “*Cronobacter*,” and “*Cedecea*.” Here, quotation marks indicate the clade names defined by those authors, rather than genus names, as each clade contains multiple genera. A total of 12 genera from that study contained at least 20 assigned genome assemblies from the Genome Taxonomy Database (GTDB), and assemblies from recognized species from these genera were analyzed using cinful. No species from the “*Cedecea*” clade were included because the two genera in this clade each contained fewer than 20 assemblies.

**FIG 7:**
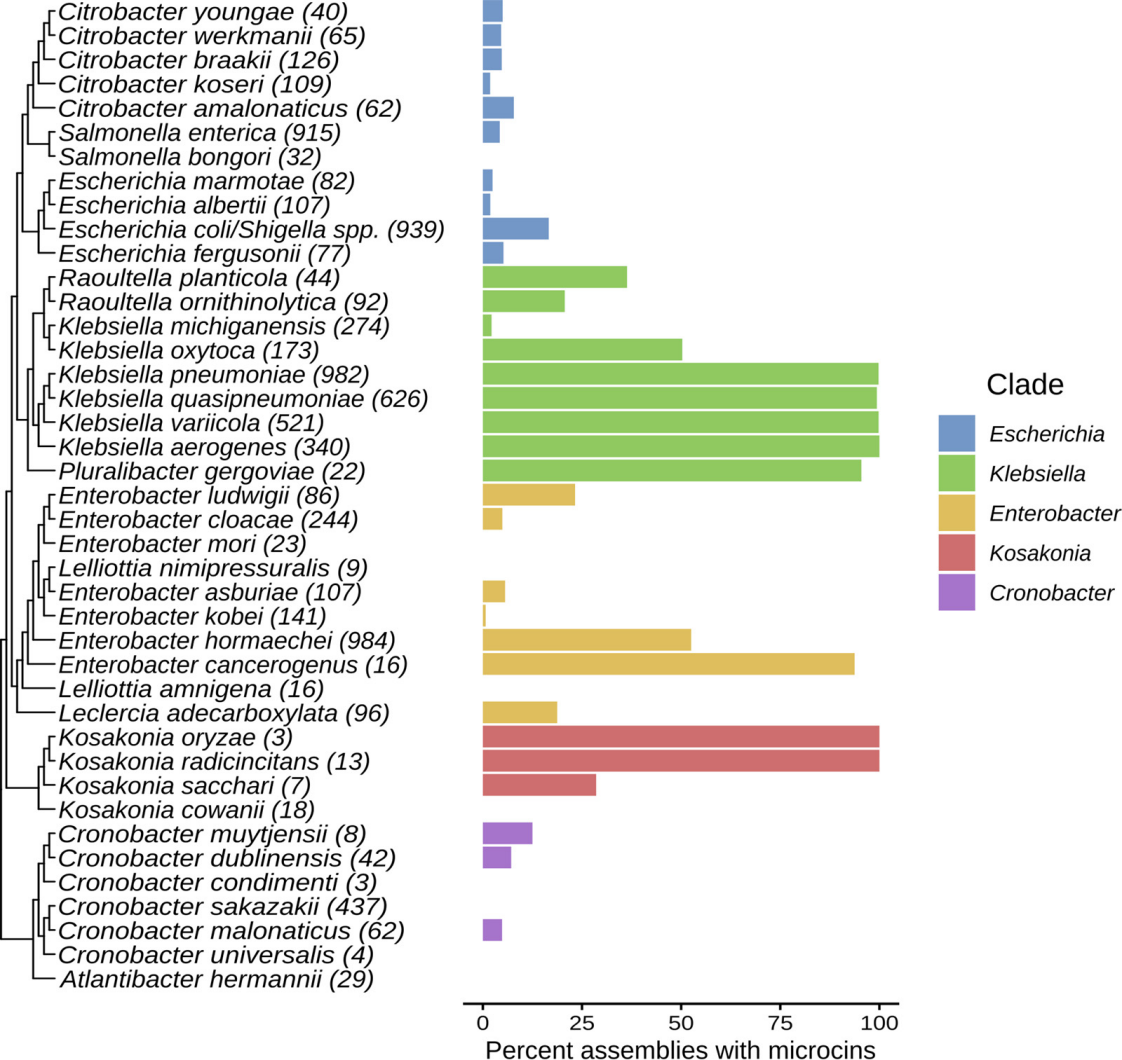
Percentage of assemblies from *Enterobacteriaceae* species with microcin homologs. The phylogenetic relationship of each species as well as the percentage of genomes in each species containing microcins are colored by their respective clade. The numbers of genomes analyzed per species are indicated in parentheses at the end of the species name. The Genome Taxonomy Database, from which taxonomy was determined, does not recognize E. coli and *Shigella* spp. as separate taxa; thus, they are represented collectively here.

The percentage of assemblies containing microcin hits varied by phylogenetic clade and by species (Fig. 7). The “Klebsiella” clade was the only clade where all species analyzed (Klebsiella spp., *Raoultella* spp., and *Pluralibacter* sp.) contained at least some assemblies with microcin hits. Furthermore, several of these “Klebsiella” clade species (Klebsiella pneumoniae, K. quasipneumoniae, K. variicola, K. aerogenes, and Pluralibacter gergoviae) contained microcins in nearly 100% of their assemblies. Among the other clades, Enterobacter cancerogenus, Kosakonia radicincitans, and Kosakonia oryzae were species where the majority of assemblies contained microcin hits. The data from this analysis are available in Supplemental File 1.

### Survey of microcins in *Enterobacterales*, *Vibrionaceae*, and *Pasteurellaceae* genomes.

**FIG 8:**
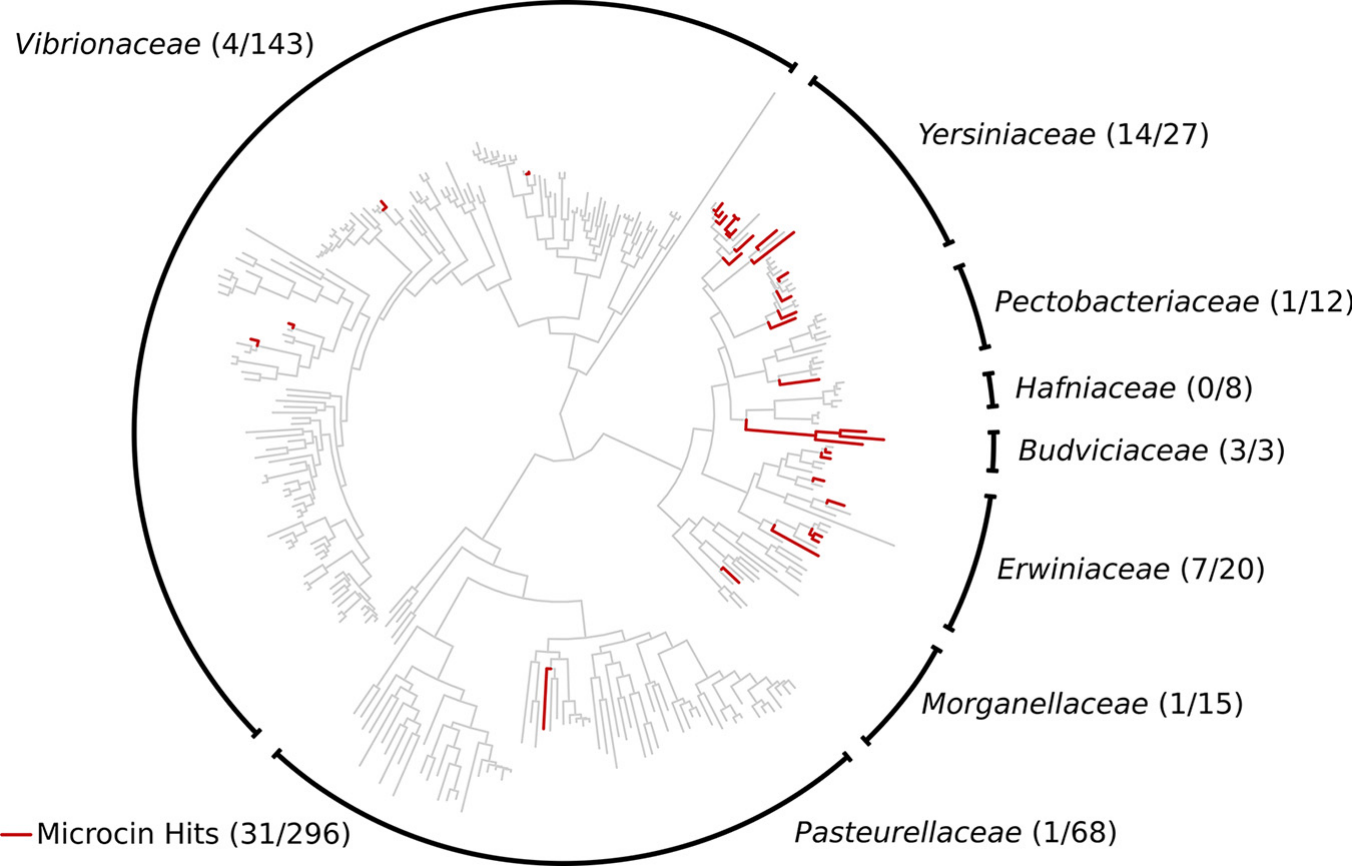
Phylogeny of *Enterobacterales*, *Vibrionaceae*, and *Pasteurellaceae* species with microcins. Each tip represents a single species, for which a single representative genome assembly was analyzed. Edges along the phylogeny with taxa that have homologs to microcins are highlighted in red. Family names are indicated for major clades. Per family, the number of species with microcins out of the total number of species sampled is indicated in parentheses. The *Enterobacteriaceae* family was not included in this analysis; it is examined in previous figures.

Current knowledge of class II microcins is limited to the *Enterobacteriaceae* family. It is unclear if class II microcins are present within other families of the *Enterobacterales* order or outside the *Enterobacterales*. An initial exploratory survey to detect microcins outside the *Enterobacteriaceae* was conducted. A single representative genome assembly from each species (*n* = 296) used in a recent phylogenomic investigation of six other families within the *Enterobacterales* (*Erwiniaceae*, *Pectobacteriaceae*, *Yersiniaceae*, *Hafniaceae*, *Morganellaceae*, and *Budviciaceae*) as well as two outgroup families (*Vibrionaceae* and *Pasteurellaceae*) was retrieved from the GTDB and provided to cinful (36). Nearly half of the species (and, thus, half of the assemblies) belonged to the *Vibrionaceae*. Results on the phylogeny from the GTDB are shown in Fig. 8. The family *Budviciaceae* contained the highest percentage of species with microcin hits (100%), although it had the fewest species sampled (*n* = 3). The *Yersiniaceae* and *Erwiniaceae* had particularly high percentages of species with microcin hits (51.9% and 40.0%, respectively), followed in microcin frequency by *Pectobacteriaceae* (8.3%) and *Morganellaceae* (6.7%). No microcins were found among representative assemblies from *Hafniaceae*. Remarkably, representative assemblies from the nonenterobacterial outgroup families contained microcin hits (*Vibrionaceae*, 2.8%; *Pasteurellaceae*, 1.5%) despite being the most distantly related taxa sampled relative to the taxa from which the 10 verified microcins originated. The data from this analysis are available in Supplemental File 1.

### Diversity of putative microcins within and beyond the *Enterobacterales*.

Among the genomes of *Enterobacteriaceae*, other *Enterobacterales*, *Vibrionaceae*, and *Pasteurellaceae* retrieved from the GTDB and analyzed by cinful (*n* = 8,340), 6,496 putative microcin hits were identified. Of these hits, 235 had 100% pairwise identity to a verified microcin; the highest number of hits was to microcin V (*n* = 79), followed by microcin E492 (*n* = 55). A total of 189 hits were exact matches to verified microcins, and all verified microcins were represented, except for N and G492. None of these exact matches to verified microcins occurred outside the *Enterobacteriaceae*, although the sampling was much sparser. In fact, only two verified microcins were found outside their *Enterobacteriaceae* clade of origin: microcin S (found in the “Enterobacter” clade) and microcin V (found in the “Klebsiella” clade).

The remaining 6,261 sequences were novel. All unique novel microcin hits (pairwise identity to a verified microcin of <100% [*n* = 425]) were aligned to their respective verified microcin to which they were a hit to view sequence diversity (Supplemental File 2B). There were more unique novel hits to class IIa microcins (*n* = 349) than to class IIb microcins (*n* = 76), with the highest number of unique novel hits being found for microcin L (*n* = 153), followed by microcin S (*n* = 78). Among the 425 unique novel hits, the average pairwise identity of the hit region to a verified microcin was 56.8%. Unsurprisingly, the average pairwise identity was the highest for putative novel microcins from members of the “Escherichia” clade (75.4%), while it was the lowest for hits from the *Vibrionaceae* family (32.8%). A total of 73 unique sequences (17.2%) had pairwise identities to a verified microcin of <40%.

**FIG 9:**
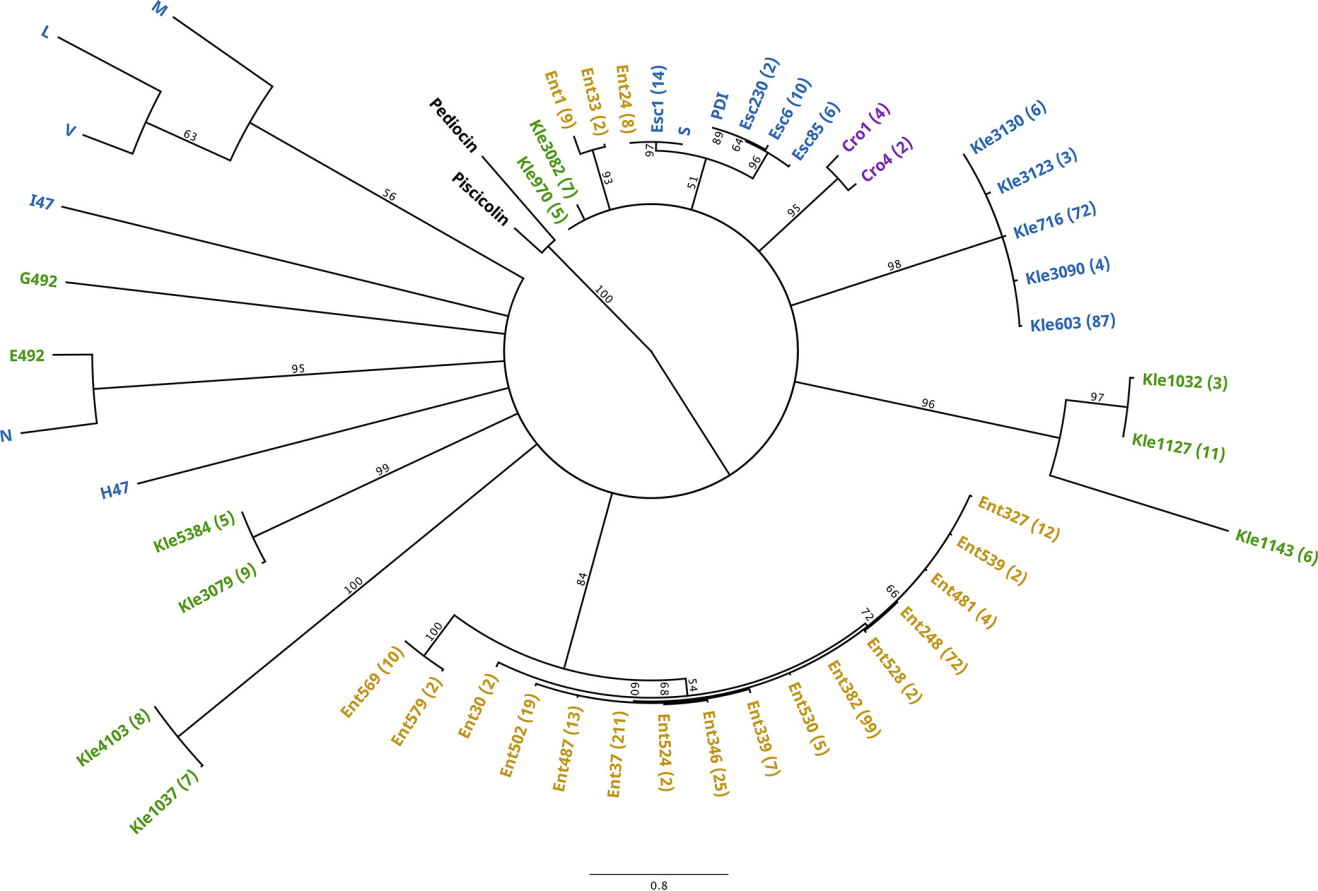
Phylogeny of putative microcins from *Enterobacteriaceae* species. A multiple-sequence alignment of cinful hits to microcins PDI and S was used to generate a maximum likelihood phylogenetic tree. All 10 verified microcins and 2 outgroup taxa (the Gram-positive double-glycine signal-containing bacteriocins pediocin PA-1 and piscicolin-126) were also included. Bootstrap support values are shown at each node, and nodes with <50% support were collapsed. Taxon identifiers are abbreviated (and color-coded) as follows for the *Enterobacteriaceae* clades from which the putative microcins were identified: Cro, “*Cronobacter*” (purple); Ent, “Enterobacter” (yellow); Esc, “Escherichia” (blue); Kle, “Klebsiella” (green). Numbers in parentheses indicate the numbers of replicate hits identified per sequence; only sequences occurring more than once were included.

To more closely examine the sequence diversity of putative microcins, we chose to generate a phylogeny of a subset of these hits. Class II microcins PDI and S have 80% pairwise identity (10), which is the highest percent identity of any pair of verified microcins. Thus, they have the potential to be homologs and share an ancestor, making them better candidates for phylogenetic analysis than more divergent microcins. Accordingly, we generated an alignment (Supplemental File 2C) and a phylogeny (Fig. 9) of 39 hits to microcins PDI and S, the 10 verified microcins for reference, and 2 Gram-positive double-glycine signal-containing bacteriocins, pediocin and piscicolin-126, as outgroup taxa. Microcins PDI and S, along with their close relatives, did resolve into the same clade but with weak support. While there is strong support for many of the subclades, the basal relationships are paraphyletic, indicating that no common ancestor could be identified (Fig. 9). This suggests that cinful has identified several novel families of microcins that are unrelated to the 10 verified microcins. Furthermore, most of these novel families (subclades) of putative microcins originate from species within the same clade of *Enterobacteriaceae*. Although this does not rule out the existence of an evolutionary relationship between microcin hits from different major evolutionary lineages of *Enterobacteriaceae*, these data suggest that microcin diversity is strongly linked to the species of origin.

An additional alignment (Supplemental File 2D) and phylogeny (Supplemental File 3) were also generated for all putative microcin hits. With the additional data, clades containing microcins PDI and S were instead paraphyletic. The tendency for microcin hits from well-supported clades to originate from taxa of similar phylogenetic origins is still evident, but more mixed sources of origin within and beyond *Enterobacteriaceae* clades can be observed in the same microcin clade, suggesting that microcin families are not strictly limited to related taxa.

## DISCUSSION

Only 10 class II microcins have been confirmed and characterized, at least in part, in terms of sequence, structure, spectrum of activity, uptake pathway, and/or mechanism of action (9, 13, 14, 23, 26, 37–51). Due to the high degree of sequence divergence, it is challenging to identify new microcins. Conversely, this high degree of sequence variation suggests that there may be a much larger pool of unsampled microcin diversity. Here, we present cinful, a bioinformatics software package to guide the discovery of class II microcins in bacterial genomic data sets. Using this pipeline, we provide evidence that both verified and novel class II microcins are widely distributed within and outside the *Enterobacteriaceae* family. We also show that the microcin prevalence varies by the habitat source of the isolate and its phylogenetic lineage.

The cinful pipeline is an improvement on other options for *in silico* class II microcin discovery in several ways. Foremost, it is developed specifically for the rapid identification of class II microcins and incorporates all currently available knowledge of verified class II microcin sequences. Previous work to search for double-glycine signal-containing peptide sequences used a profile HMM (pHMM) trained only on the signal sequences (31), excluding the core peptide sequences. These putative signal sequences were mostly from proteins of unknown function, although signals from three verified microcins were included (32). Other leading software programs for general bacteriocin detection, such as BAGEL4 (52), use methods similar to those of cinful but produce an output that requires the time-intensive visualization of gene clusters to manually identify bacteriocins. In order to conduct a global screen focused on class II microcins, the development of a new, more precise, and high-throughput method was necessary.

With cinful, the microcin pHMM was trained exclusively with confirmed class II microcins, inclusive of the entire precursor peptide, with the goal of improving the identification of confirmed microcin homologs. Furthermore, the training set included a precisely curated collection of all 10 verified microcins, only subsets of which are represented in previous pipelines (31, 52). Second, we sought to incorporate the identification of genes specific to class II microcins by screening for the associated PCAT, MFP, and immunity protein, again using a curated collection of verified proteins. This step provides added confidence that a candidate peptide is a canonical microcin. Third, we constructed our analysis around the goal of providing an easily parsed output file containing data on putative microcins and other identified genes. In concert, these improvements have resulted in a rapid, verifiable, high-throughput screening method built within a modern and reusable pipeline that is conducive to downstream analysis. Although our study is focused on class II microcins, we can imagine that a class I microcin-specific pipeline could be designed according to similar guidelines, as we believe that a one-size-fits-all bacteriocin finder is less likely to effectively identify novel microcins.

In addition to identifying putative microcins, our bioinformatics pipeline includes identification of the associated immunity proteins and export machinery (PCATs and MFPs). Specific to the PCAT, our understanding of the C39 peptidase domain has been clarified: we now know the highly homologous C39-like peptidase domain lacks the requisite protease activity to cleave the signal sequence (53). In a previous study by Dirix et al. (31), prospective PCAT hits were manually inspected for the presence of two peptidase domain motifs, which incidentally included all catalytic triad residues. However, these motifs may be unnecessarily restrictive, as the three residues alone have now been experimentally shown to be essential for protease activity (54, 55). Our analysis of E. coli genome assemblies highlights the current challenges in identifying these components. In an ideal world, all of these components would be neatly organized (i.e., encoded adjacent to each other) on the genome or plasmid assembly and easy to identify (56). In reality, this is not entirely the case for at least two reasons. First, even if these components are neatly organized and easy to identify, the genome assembly quality greatly affects the ability to identify them. For example, since PCATs and MFPs are large proteins, fragmented assemblies may not have enough coverage to identify the gene. Second, immunity proteins provide additional challenges to identification. Because the 10 verified immunity proteins show no obvious sequence similarity and have a broader range of sizes than the microcins, it is difficult to know if our current search parameters are optimal. Further characterization of newly identified microcins and their associated components will provide better insights into microcin genetic architecture, from which better identification methods can be developed. Due to these challenges, the analysis of microcins beyond E. coli focused on the presence of putative microcin sequences only.

This pipeline was used to demonstrate that nearly one-quarter of the genomes from E. coli may be microcinogenic. This supports a similar estimate from the Antimicrobial Peptide Database (57) that shows that 34.1% of E. coli strains contain microcins (18). Microcins are widespread among the *Enterobacteriaceae*, particularly among the “Klebsiella” clade. Furthermore, even though our sampling among *Enterobacterales* was sparse, with only one representative genome being sampled per species, microcins appear to be distributed among most of the other families of *Enterobacterales*, particularly among members of the *Budviciaceae*, *Yersiniaceae*, and *Erwiniaceae*. Despite the sparse sampling, restriction to representative genomes, and the use of software trained on *Enterobacteriaceae*-derived microcins, we provide the first systematic evidence of putative microcins in the *Vibrionaceae* and *Pasteurellaceae*, the two gammaproteobacterial families most closely related to, but outside, the *Enterobacterales* (58). One could speculate that even if true microcin homologs exist in phylogenetically disparate taxa, their sequence divergence may covary with increasing phylogenetic distance sufficiently to avoid detection. This hints that microcins exist beyond the *Enterobacterales*, which warrants a wider investigation. If verifiable, such microcins would have interesting implications regarding microcin evolutionary history and ecology.

Although there is still much to be learned about microcin biology, we can now begin to develop an understanding of the roles that genotype and habitat play in shaping the patterns of microcin diversity found in nature. This analysis highlights patterns in the variation of class II microcin diversity between different phylogroups. Besides their phylogenetic delineation, isolates from the same phylogroup share other genetic signatures (e.g., genome size and virulence factors) (33, 59). The prevalence of E. coli phylogroups is known to vary by host as well as bacterial lifestyle (e.g., commensal versus pathogen status) (33, 60). The results here suggest that E. coli isolates from phylogroups G and B2 are particularly enriched for microcins. This aligns with results from a PCR-based screen of human fecal isolates for microcin determinants, which showed enrichment in phylogroup B2 compared to the other major (most abundant) phylogroups (A, B1, and D) (61). The results also suggest that specific microcins are enriched in particular phylogroups. For example, the enrichment/depletion patterns of specific microcin homologs in phylogroups B2 and A show an interesting contrast. Phylogroup B2 is enriched with class IIb microcins M and H47 and depleted/neutral for other microcins. Phylogroup A is enriched for class IIa microcin V and, to a lesser extent, the other class IIa microcins while being depleted for the class IIb microcins. Given the different lifestyle/habitat characteristics of these phylogroups (phylogroup A is associated with human commensal strains [62], and phylogroup B2 is associated with inflammatory bowel disease [63] and extraintestinal infections [64, 65]), one could speculate that the carriage of specific microcins provides an adaptive advantage in specific lineages/habitats.

Among the different habitats sampled here, E. coli isolates from freshwater sources are significantly depleted for microcins, suggesting that E. coli isolates from host-associated habitats more frequently contain microcins. Similarly, in the original study of this E. coli data set, fewer colicins, a class of larger bacteriocins produced by E. coli, were found among isolates from the predominant freshwater phylogroup, B1, than among isolates from the most underrepresented freshwater phylogroup, B2 (33). The caveat here is that our microcin search is predicated upon the sequences of host-derived, verified microcins; none of the verified microcins are from isolates of freshwater origin. E. coli isolates from human extraintestinal sources were the most enriched for microcins. Similarly, when comparing microcin determinants from the above-mentioned PCR-based screen of fecal samples, a follow-up study found greater carriage of microcin determinants in human extraintestinal E. coli isolates than in those fecal isolates (66). Furthermore, E. coli isolates from poultry meat were also enriched. Although there is some suggestion that poultry may be a source of human extraintestinal pathogenic E. coli infections (67, 68), the commonality between human extraintestinal and poultry meat isolates regarding microcin carriage remains to be determined. The specific process that allows microcins to proliferate in a population is still unclear, but it seems likely that the isolate habitat plays an important role. It should be noted that the E. coli isolate assemblies that were analyzed in this study are all sourced from Australia. Although several different habitats are represented, many other habitats of potential interest were absent. The microcin distributions found among these isolates may not be representative of those in other geographic locations and habitats.

A caveat of our study is that the number of confirmed microcins included in the training set is still relatively small, and the detection of novel microcins would likely be improved with a larger, more diverse data set. The availability of sequence data is another limitation that we have attempted to minimize through the careful selection of diverse genomic data sets. The detection of novel microcins with cinful relies on the query protein having a BLAST hit to one of the 10 verified class II microcins, which is inherently limiting. This is likely particularly true for species that are more distantly related to E. coli and K. pneumoniae, as microcins are generally understood to be active primarily toward (and, thus, produced by) more closely related strains (18). We anticipate that this first collection of hits can be screened *in vitro* for antibacterial activity, and those with confirmed activity and other associated features of canonical microcins can be included in the training set to improve the detection of a broader range of microcins and their associated components.

In addition to the limitations of detecting the microcin precursor peptides, certain assumptions involved in the detection of the PCATs, MFPs, and candidate immunity proteins may be too restrictive or permissive. For example, given that the largest known immunity protein (belonging to microcin S) is an outlier at 216 amino acids, we selected a maximum size cutoff of 250 amino acids. However, there could be novel immunity proteins larger than this that have not yet been identified, or perhaps not all microcins have specific immunity proteins. Furthermore, multiple microcins can be present in the same genome, as demonstrated by our analyses, and certain microcins in particular tend to cooccur (69).

Much of the current knowledge of microcins is limited to two species. Here, we provide evidence that class II microcins are widespread throughout other *Enterobacteriaceae* species as well as representative species of other families within the *Enterobacterales* and beyond. From this analysis, we cannot pinpoint the evolutionary origins of microcins. Since there is no clear direct association between evolutionary relationships and the occurrence of class II microcins, horizontal gene transfer may play an important role in the patterns of widespread class II microcins observed here in the *Enterobacterales*. In fact, a recent large-scale analysis of the biogeography of prokaryotic proteins in metagenomic assemblies determined that while most species-level genes are associated with a single, specific habitat, the small percentage of genes that occur in multiple habitats proliferate via horizontal gene transfer (70). The identification of new and unique microcins will allow questions about the evolution and function of this class of bacterial peptides to be addressed in greater detail.

## MATERIALS AND METHODS

### Pipeline development.

**(i) Pipeline overview.** The cinful pipeline is developed as a Python package, and third-party software dependencies and libraries used in the pipeline are freely distributed through Anaconda.

**(ii) Functionally validated sequence data set.** The class II microcin training data set was composed of the precursor peptides (i.e., signal sequence plus core peptide) of the 10 verified class II microcins with confirmed antibacterial activity. These include class IIa microcins V (26), L (71), N (72), PDI (48), and S (46) and class IIb microcins H47 (45), I47 (73), M (39), E492 (24), and G492 (37). The immunity protein training data set was formed from the cognate immunity proteins of each of these microcins. The PCAT and MFP training data sets were sourced from the 10 verified microcins, but in this case, 2 pairs of microcins cooccur in the same bacterial strain and share an export system (37), resulting in 8 sets of a PCAT and an MFP.

**(iii) Protein-coding gene identification.** Protein-coding genes were identified in input genomic scaffolds using Prodigal v2.6.3 with metagenomic settings (74), recently benchmarked as the best coding sequence predictor for E. coli (75). To improve the downstream search efficiency, a protein database, which was nonredundant on a per-sample basis, was generated from all inferred protein sequences from Prodigal. The sequences were given a unique identifier using the seqhash algorithm (76), which was used to record the sample, contig identifier, and coordinates for all redundant protein sequences. This information was used later to cross-reference the various contigs from which each unique peptide sequence was inferred.

**(iv) Microcin identification.** Currently verified class II microcins range in size from 75 to 120 amino acids (for the precursor peptides, including their signal sequences). To prevent needlessly searching peptide sequences that were much larger than what is expected for class II microcins, only sequences within 30 to 150 amino acids in length were retained using seqkit v0.15.0 (77). High sensitivity is preferred for the identification of microcins due to their high sequence divergence. Class II microcin homologs were identified in the input data set using a combination of BLAST v2.9.0 (blastp –evalue 0.001) and HMMER v3.3.1 using default settings (78, 79). First, protein sequences of the 10 verified class II microcins were used to generate a BLASTP database against which the size-delimited protein sequences from the input data set were queried. BLASTP hits were then analyzed using a profile hidden Markov model (pHMM) to yield the final putative microcin hits. The pHMM was constructed using the 10 verified microcin amino acid sequences by generating a multiple-sequence alignment (MSA) with MAFFT v7.475 using the –auto setting (80). The MSA was then converted to a pHMM using hmmbuild from HMMER. The alignment also allowed us to assess the sequence homology of the 10 class II microcins and was visualized using Geneious Prime 2022.1.1 (https://www.geneious.com). The phylogenetic relationships of the class II microcins were reconstructed using an approximate maximum likelihood framework with FastTree v2.1 using default settings (81) and visualized using the phylogram package in R (https://CRAN.R-project.org/package=phylogram).

**(v) PCAT and MFP identification.** PCATs and MFPs have much lower sequence divergence than microcins, which allowed the use of DIAMOND v2.0.11 with –evalue 0.001 in place of BLAST in order to improve the speed (82). For PCATs, only query proteins within 600 to 800 amino acids in size were selected for the homology search. For MFPs, this size restriction was 375 to 450 amino acids. The results were then further filtered to allow only hits that have sufficient overlap with true PCATs or MFPs. This was achieved by aligning the putative hits with the initial MSA using the –add feature of MAFFT, and only residues that lined up with the original verified sequences were retained using the –keeplength feature. Sequences that were composed of more than 10% gaps and more than 10% residues that did not line up with the original alignment were not retained for downstream analysis. Additionally, mutational studies of the PCAT that exports microcin V, CvaB, identified three conserved residues that act as a catalytic triad to maintain the proteolytic functionality necessary for signal cleavage and microcin export (54, 55). Thus, only PCAT hits with these residues (Cys32, His105, and Asp121, according to the CvaB reference sequence) in the respective homologous positions were kept.

**(vi) Immunity protein candidate identification.** Identifying immunity proteins using sequence homology is difficult as there is little to no sequence similarity among the currently verified immunity proteins. Our initial search was conducted using a combination of BLAST and HMMER trained on the 10 known immunity proteins, resulting in a single candidate per contig. However, many of the hits identified were uncharacteristic of known immunity proteins due to the genomic location being distant from the putative microcin; these hits also had low percent identities to the known immunity proteins. As a solution, we made use of the known characteristics of previously investigated immunity proteins to identify a list of the best candidates. First, confirmed immunity proteins range between 51 and 216 amino acids in length (23, 46). Second, immunity proteins are in proximity to the microcin (83). Third, immunity proteins tend to be located on the inner membrane and have one or more transmembrane helical domains (23, 38). Thus, the three closest protein-coding genes up- and downstream (total of six proteins) of the best microcin hit per contig were screened for length; sequences with 30 to 250 amino acids were retained. Those within the length cutoff were considered immunity protein candidates and were provided as the input to pyTMHMM v1.3.2 to predict the presence of transmembrane helical domains (84). The presence of transmembrane helical domains is not treated as a screening criterion but may provide additional information for determining immunity proteins from the identified candidates.

**(vii) Output files.** Once putative microcins, PCATs, and MFPs, as well as the candidate immunity proteins, were identified, the final step in the cinful pipeline is reporting the results of the search. Three files are output: the first file contains the highest-percent-identity amino acid sequences identified as a BLAST hit per contig for each component, including the microcin, MFP, PCAT, and immunity proteins. The second file contains the characteristic search for immunity protein candidates, including the potential for up to six immunity protein candidates per putative microcin. The third file contains each contig that contains a microcin, MFP, and PCAT. These files are contained in a single output folder denoted the “best hits.”

### Survey of microcins in the E. coli pangenome.

Microcin prevalence was analyzed in genome assemblies from host-associated and freshwater E. coli isolates from Australia (BioProject accession number PRJEB34791) (33). Of the 1,924 assemblies produced by Touchon et al. (33), a total of 1,224 were successfully retrieved with ncbi-genome-download v0.3.1 using the NCBI genome accession numbers. Strains were categorized by Touchon et al. into four broad source categories: human, nonhuman mammal, bird, and environmental. Of the 1,224 retrieved assemblies, human sources included extraintestinal (from urine or blood) (*n* = 104), intestinal (from biopsy specimens from healthy or inflamed intestines) (*n* = 172), and fecal (from healthy hosts) (*n* = 72) sources. Nonhuman mammal sources included only fecal sources (*n* = 117). Bird sources included meat (*n* = 283) and fecal (*n* = 185) sources. Environmental sources included only freshwater sources (*n* = 280). An additional 11 isolates did not fall into any of these seven subcategories. The E. coli phylogroups represented by this data set were phylogroups A (*n* = 292), B1 (*n* = 278), B2 (*n* = 304), C (*n* = 16), D (*n* = 174), E (*n* = 57), F (*n* = 71), and G (*n* = 32). These 1,224 assemblies were provided as the input to our bioinformatics pipeline to determine the following: (i) how widespread microcins are in E. coli, (ii) putative novel microcins in E. coli, and (iii) whether microcins are enriched or depleted in assemblies originating from different phylogroups or source categories. The above-mentioned 11 isolates with no source subcategory were excluded from source-based analyses. Phylogenetic relationships of E. coli phylogroups were inferred from a recent phylogenomic analysis of >10,000 E. coli genomes (59).

### Survey of microcins in *Enterobacteriaceae* genomes.

To determine the extent to which microcins exist among the *Enterobacteriaceae*, we strategically retrieved genome assemblies from different species in this family to provide as the input to cinful. A recent phylogenomic analysis of 76 species belonging to 26 genera from the *Enterobacteriaceae* identified six separate clades (“Escherichia,” “Klebsiella,” “Enterobacter,” “*Kosakonia*,” “*Cronobacter*,” and “*Cedecea*”) (35). That analysis used one or two representative genome assemblies from each species, but our goal was to determine how prevalent class II microcins are throughout numerous isolates across each species in each of the separate clades. To do this, we retrieved all available genome assemblies that have been assigned to each of these species with the Genome Taxonomy Database (GTDB) (85), provided that there were at least 20 genome assemblies per genus. The exceptions to this were the species E. coli, K. pneumoniae, Salmonella enterica, and Enterobacter hormaechei. These species have thousands of genome assemblies available, so 1,000 accession numbers were randomly subsampled from the GTDB for each species, and available assemblies from these accession numbers were retrieved. The proportion of assemblies with microcin hits was mapped onto the phylogenetic topology inferred by Alnajar and Gupta (35).

### Survey of microcins in *Enterobacterales*, *Vibrionaceae*, and *Pasteurellaceae* genomes.

A search for microcins outside the *Enterobacteriaceae* was conducted. A recent phylogenomic study of 179 representative members of the *Enterobacterales* delineated seven monophyletic families (*Enterobacteriaceae*, *Erwiniaceae*, *Pectobacteriaceae*, *Yersiniaceae*, *Hafniaceae*, *Morganellaceae*, and *Budviciaceae*), with representatives from *Pasteurellaceae* and *Vibrionaceae* as the outgroups (36). To determine the extent to which microcins occur throughout other families of the *Enterobacterales*, one genome assembly from each recognized species used in the phylogenomic analysis by Adeolu et al. (36) (excluding species of *Enterobacteriaceae*) was retrieved from the GTDB, selecting only the assemblies designated “GTDB representative of species.” Additionally, genome assemblies from the same outgroup families (*Pasteurellaceae* and *Vibrionaceae*) as the ones used by Adeolu et al. were retrieved from the GTDB by identifying every species with a genome assembly designated “GTDB representative of species,” yielding one genome assembly per species. These assemblies were provided as the input to our bioinformatics pipeline, and the species with microcin hits in their representative genomes were highlighted on the phylogenetic topology provided by the GTDB.

### Alignments of novel microcins.

MSAs of novel microcins identified from both the E. coli study data set (33) and the *Enterobacterales*, *Vibrionaceae*, and *Pasteurellaceae* genomes retrieved from the GTDB by us were generated using MAFFT (80). Alignments were produced with the verified microcin to which the novel microcins were a hit as the reference sequence per alignment. Only hits with a pairwise identity of <100% to the verified microcin were included, and replicate sequences were deduplicated prior to alignment and represented by a single sequence identifier. Abbreviations used for the sequence identifiers for the *Enterobacteriaceae* clades are Cro for “*Cronobacter*,” Ent for “Enterobacter,” Esc for “Escherichia,” and Kle for “Klebsiella,” and those for other *Enterobacterales* families are Bud for *Budviciaceae*, Erw for *Erwiniaceae*, Mor for *Morganellaceae*, Pec for *Pectobacteriaceae*, Yer for *Yersiniaceae*, Pas for *Pasteurellaceae*, and Vib for *Vibrionaceae* (Supplemental Files 2B, 2C, 2D).

### Putative *Enterobacteriaceae* microcin phylogeny.

An MSA of cinful hits to microcins PDI and S from the *Enterobacteriaceae*, as identified by our analysis of genomes from the GTDB, was produced with MAFFT (80). Hits lacking valid start or stop codons (i.e., protein fragments), without a recognizable signal cleavage site, containing ambiguous characters (e.g., X), or of <50 amino acids in size were excluded from the MSA. For each *Enterobacteriaceae* clade, only hits with more than one occurrence were included, with duplicates being removed before the MSA was generated. All 10 verified microcins were included for comparison. The Gram-positive bacteriocins pediocin (86) and piscicolin-126 (87), which also have a double-glycine signal sequence, were included as the outgroup. A maximum likelihood phylogenetic tree was generated from this alignment using RAxML (88). The Whelan and Goldman (WAG); https://academic.oup.com/mbe/article/18/5/691/1018653 model of protein evolution was used, and 1,000 bootstrap replicates were generated. The same procedures were performed to produce an MSA and phylogeny of all cinful microcin hits from all GTDB genomes (including taxa outside the *Enterobacteriaceae*), with the exception that hits with a single occurrence were included.

### Data availability.

All data and analysis scripts for this work are available at https://github.com/wilkelab/cinful_data_analysis. Statistical analysis and plotting were done in R (89) using the tidyverse family of packages (90). The genome accession numbers for assemblies used in this analysis and the output files from cinful are provided in Supplemental File 1 as well as the repository mentioned above. All assemblies used were downloaded from the NCBI GenBank database using https://github.com/kblin/ncbi-genome-download. The source code for our bioinformatics pipeline cinful is available at https://github.com/wilkelab/cinful.
