# Supplementary material for: Evidence for Widespread Class II Microcins in Enterobacterales Genomes
Source: Appl Environ Microbiol. 2022 Nov 17;88(23):e01486-22. doi: 10.1128/aem.01486-22 (PMC9746304; doi:10.1128/aem.01486-22)
Supplement: Supplemental file 2 — Fig. S2 to S5. Download aem.01486-22-s0002.pdf, PDF file, 14.1 MB [file aem.01486-22-s0002.pdf]

Supplemental File 2A. Multiple sequence alignments of novel microcin sequences identified by *cinful* from E. coli assemblies. Unique putative microcin sequences with pairwise identities of <100% to the verified microcin to which they were a hit are shown (n=40). Per alignment, the verified microcin is shown at the top as a reference sequence, with all of its associated hits shown below. Disagreements to the reference sequence are highlighted. Alignments were generated using MAFFT and visualized in Geneious Prime.

Class IIa Novel Microcin Hits

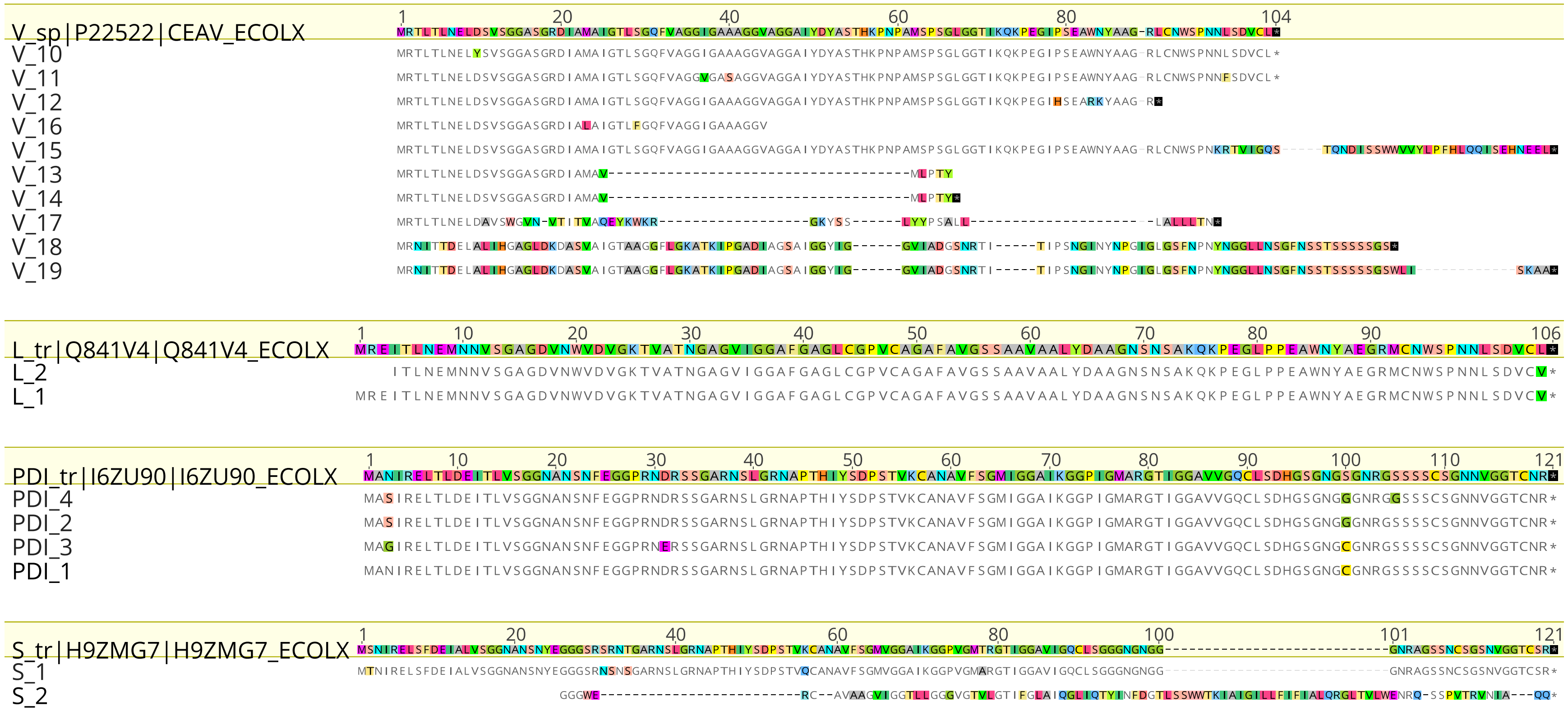

Class IIb Novel Microcin Hits

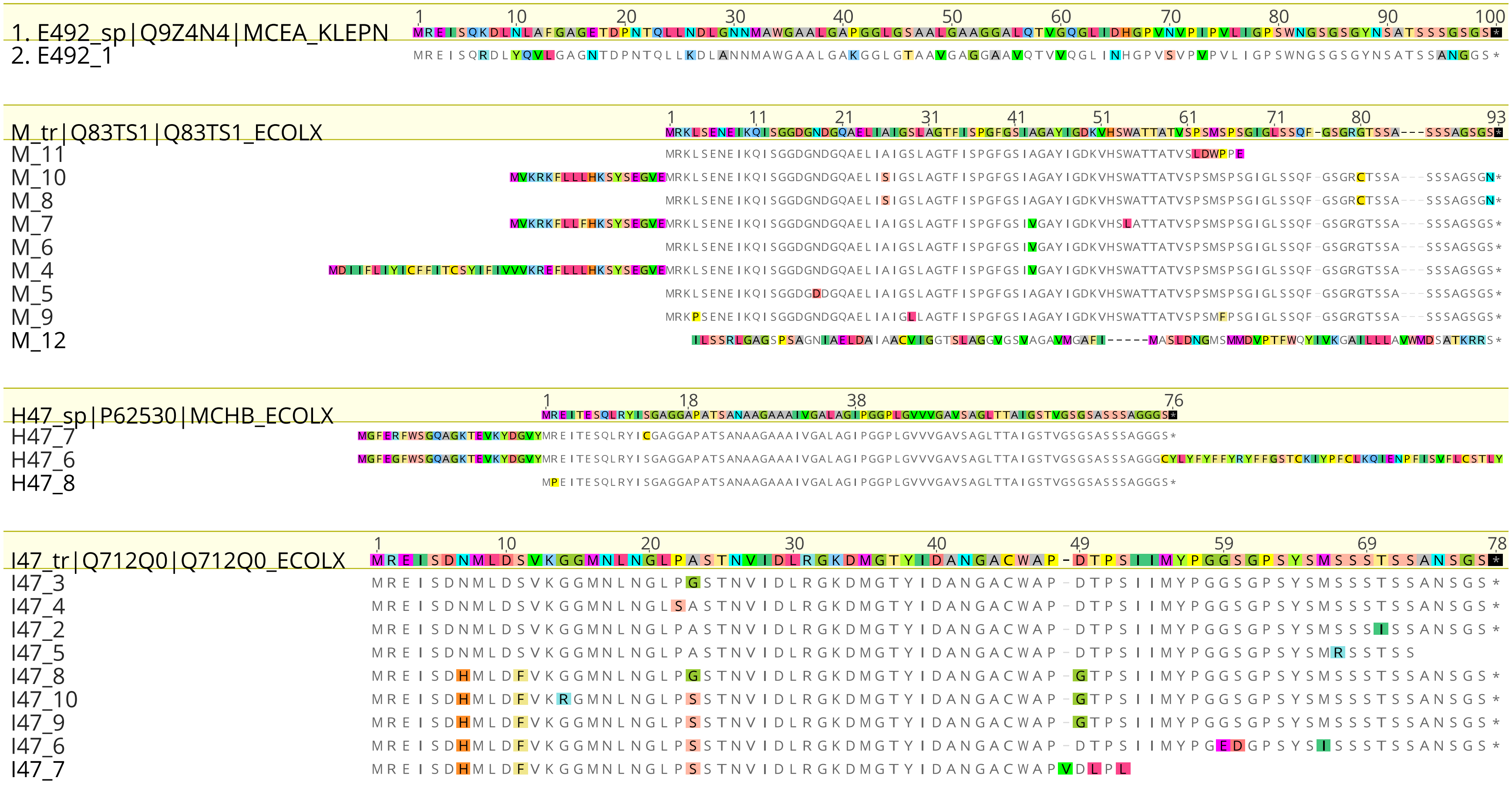

Supplemental File 2B. Multiple sequence alignments of novel microcin sequences identified by *cinful* from Enterobacterales, *Vibrionaceae*, and *Pasteurellaceae* assemblies. Unique putative microcin sequences with pairwise identities of <100% to the verified microcin to which they were a hit are shown (n=425). Per alignment, the verified microcin is shown at the top as a reference sequence, with all of its associated hits (with number of replicates in parentheses) shown below. Disagreements to the reference sequence are highlighted. Alignments were generated using MAFFT and visualized in Geneious Prime.

Class IIa Novel Microcin Hits

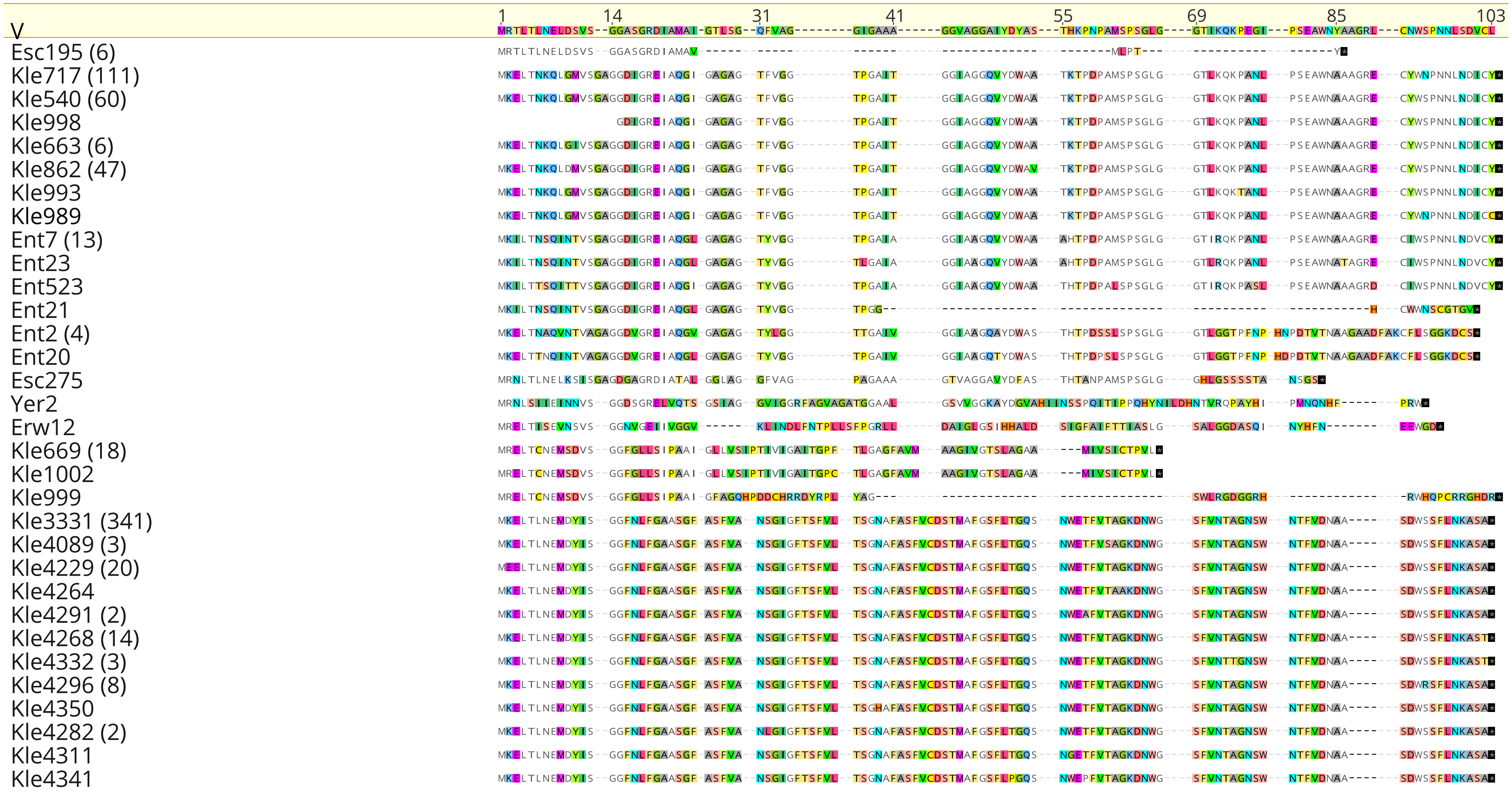

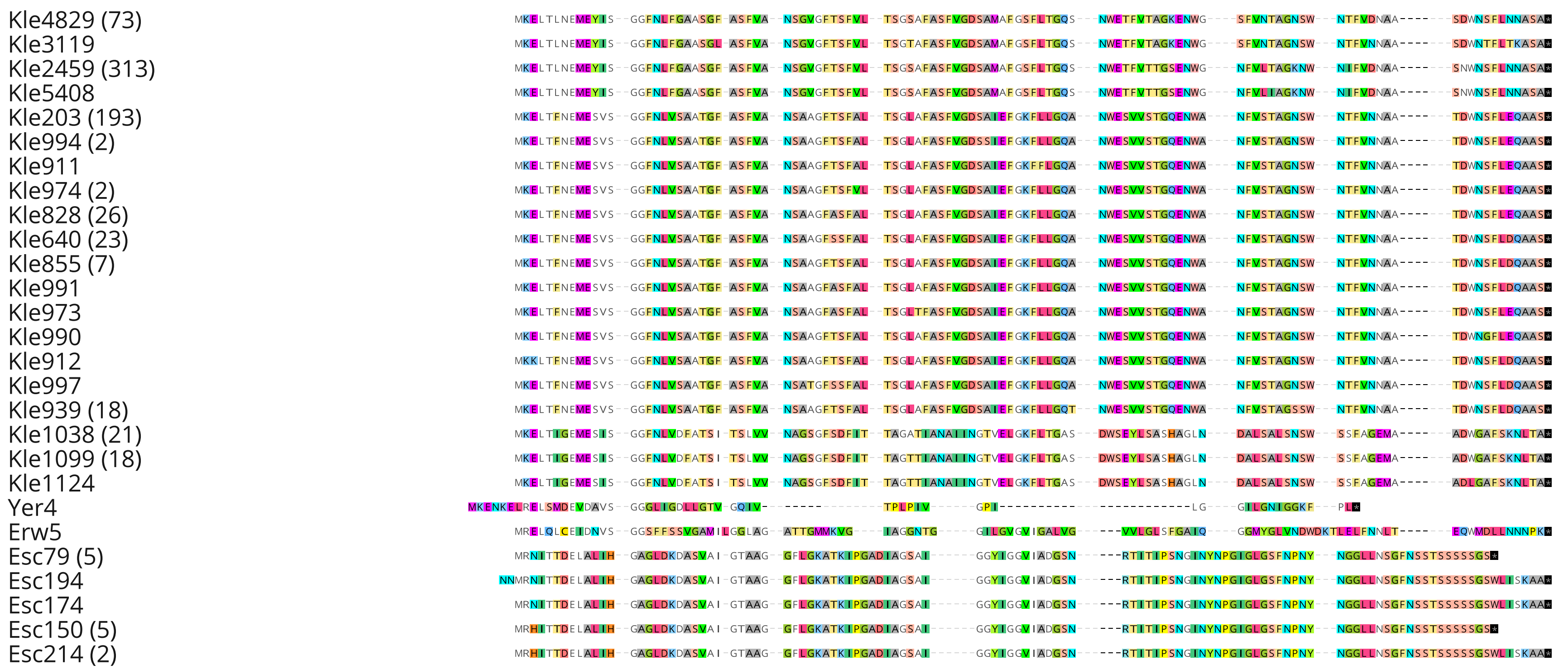
Kle4829 (73)
Kle3119
Kle2459 (313)
Kle5408
Kle203 (193)
Kle994 (2)
Kle911
Kle974 (2)
Kle828 (26)
Kle640 (23)
Kle855 (7)
Kle991
Kle973
Kle990
Kle912
Kle997
Kle939 (18)
Kle1038 (21)
Kle1099 (18)
Kle1124
Yer4
Erw5
Esc79 (5)
Esc194
Esc174
Esc150 (5)
Esc214 (2)

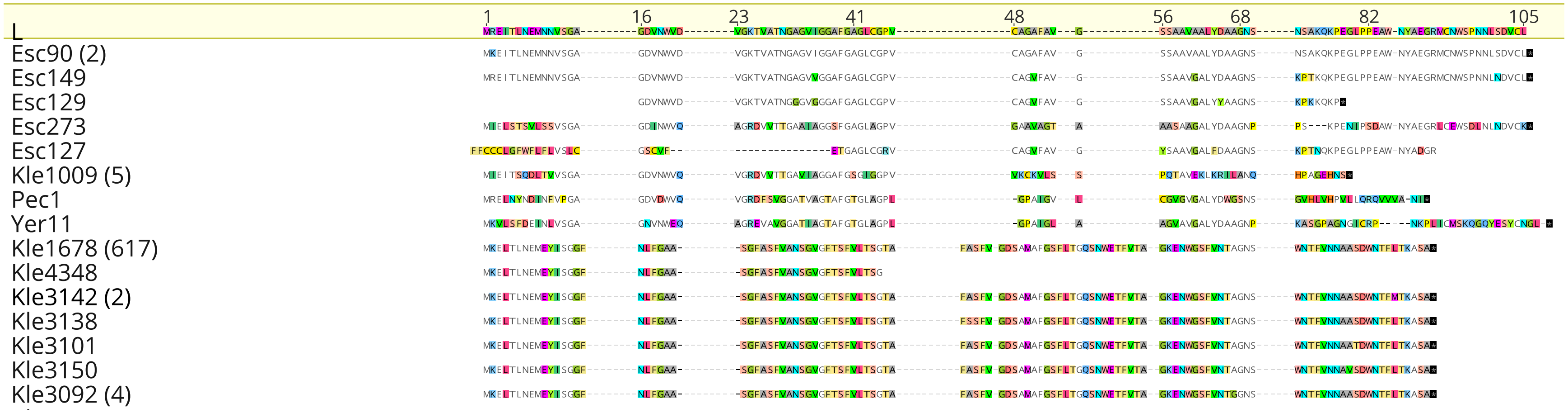
1
16
23
41
48
56
68
82
105
L
Esc90 (2)
Esc149
Esc129
Esc273
Esc127
Kle1009 (5)
Pec1
Yer11
Kle1678 (617)
Kle4348
Kle3142 (2)
Kle3138
Kle3101
Kle3150
Kle3092 (4)

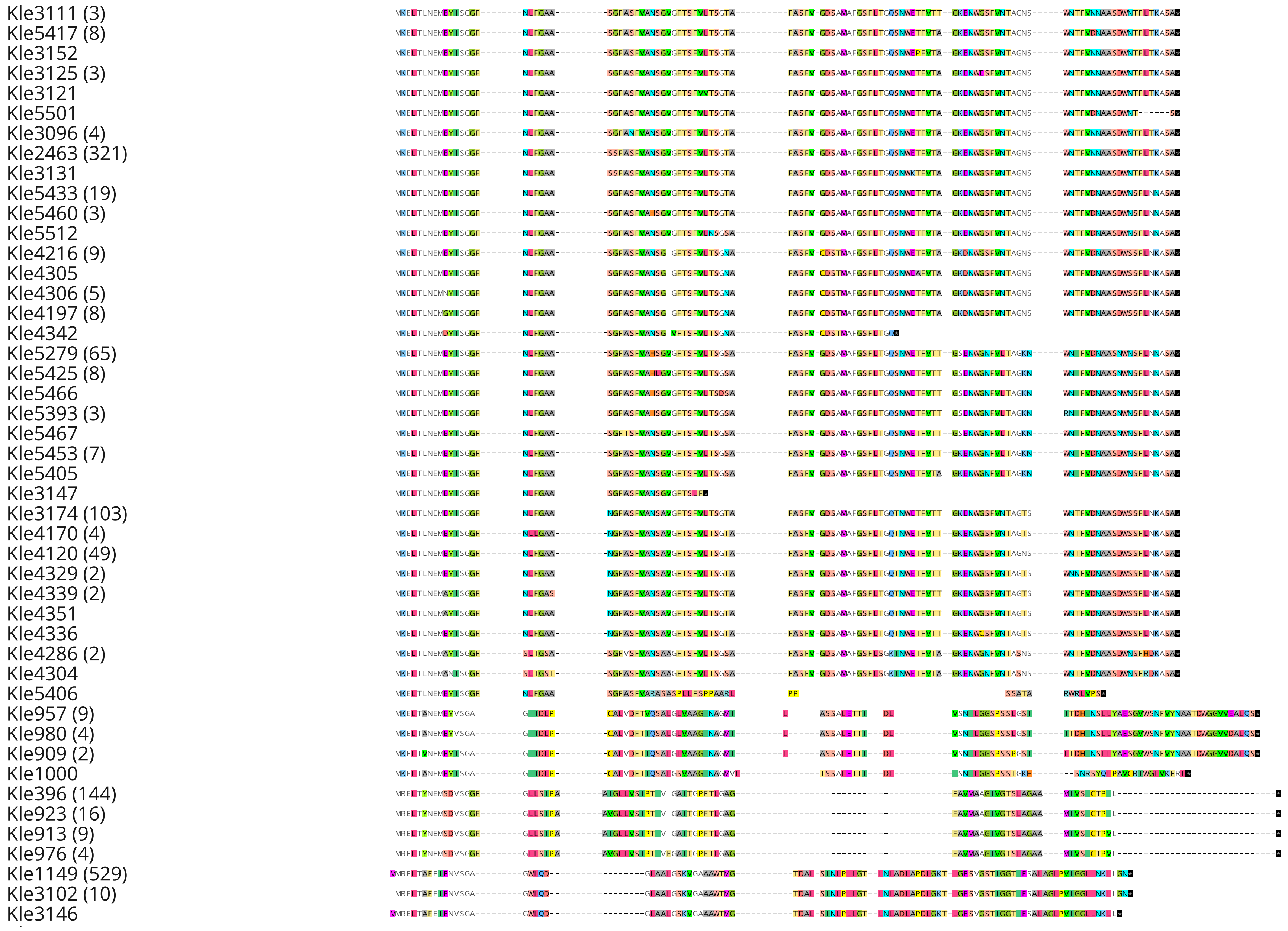
Kle3111 (3)
Kle5417 (8)
Kle3152
Kle3125 (3)
Kle3121
Kle5501
Kle3096 (4)
Kle2463 (321)
Kle3131
Kle5433 (19)
Kle5460 (3)
Kle5512
Kle4216 (9)
Kle4305
Kle4306 (5)
Kle4197 (8)
Kle4342
Kle5279 (65)
Kle5425 (8)
Kle5466
Kle5393 (3)
Kle5467
Kle5453 (7)
Kle5405
Kle3147
Kle3174 (103)
Kle4170 (4)
Kle4120 (49)
Kle4329 (2)
Kle4339 (2)
Kle4351
Kle4336
Kle4286 (2)
Kle4304
Kle5406
Kle957 (9)
Kle980 (4)
Kle909 (2)
Kle1000
Kle396 (144)
Kle923 (16)
Kle913 (9)
Kle976 (4)
Kle1149 (529)
Kle3102 (10)
Kle3146

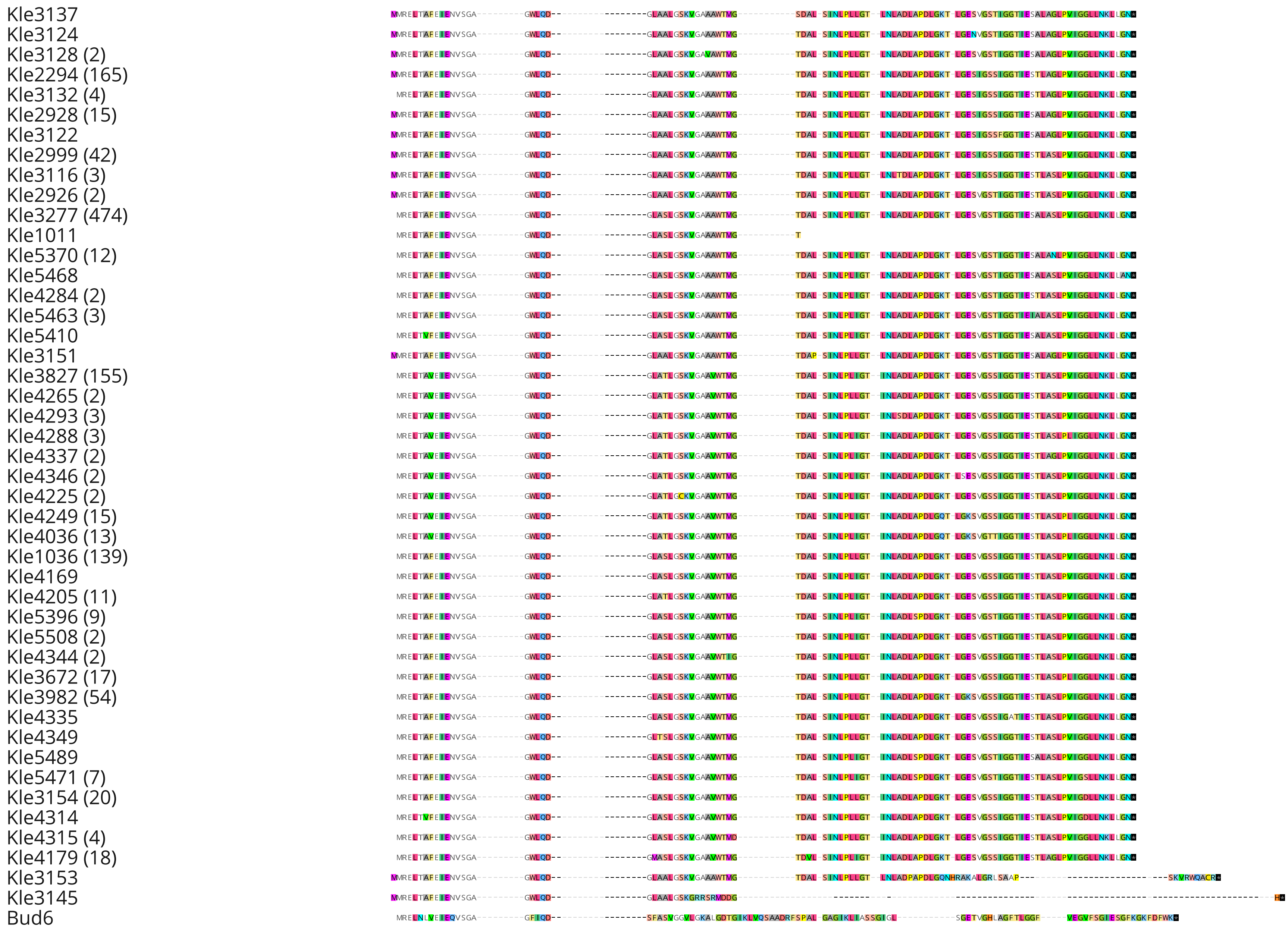
Kle3137
Kle3124
Kle3128 (2)
Kle2294 (165)
Kle3132 (4)
Kle2928 (15)
Kle3122
Kle2999 (42)
Kle3116 (3)
Kle2926 (2)
Kle3277 (474)
Kle1011
Kle5370 (12)
Kle5468
Kle4284 (2)
Kle5463 (3)
Kle5410
Kle3151
Kle3827 (155)
Kle4265 (2)
Kle4293 (3)
Kle4288 (3)
Kle4337 (2)
Kle4346 (2)
Kle4225 (2)
Kle4249 (15)
Kle4036 (13)
Kle1036 (139)
Kle4169
Kle4205 (11)
Kle5396 (9)
Kle5508 (2)
Kle4344 (2)
Kle3672 (17)
Kle3982 (54)
Kle4335
Kle4349
Kle5489
Kle5471 (7)
Kle3154 (20)
Kle4314
Kle4315 (4)
Kle4179 (18)
Kle3153
Kle3145
Bud6

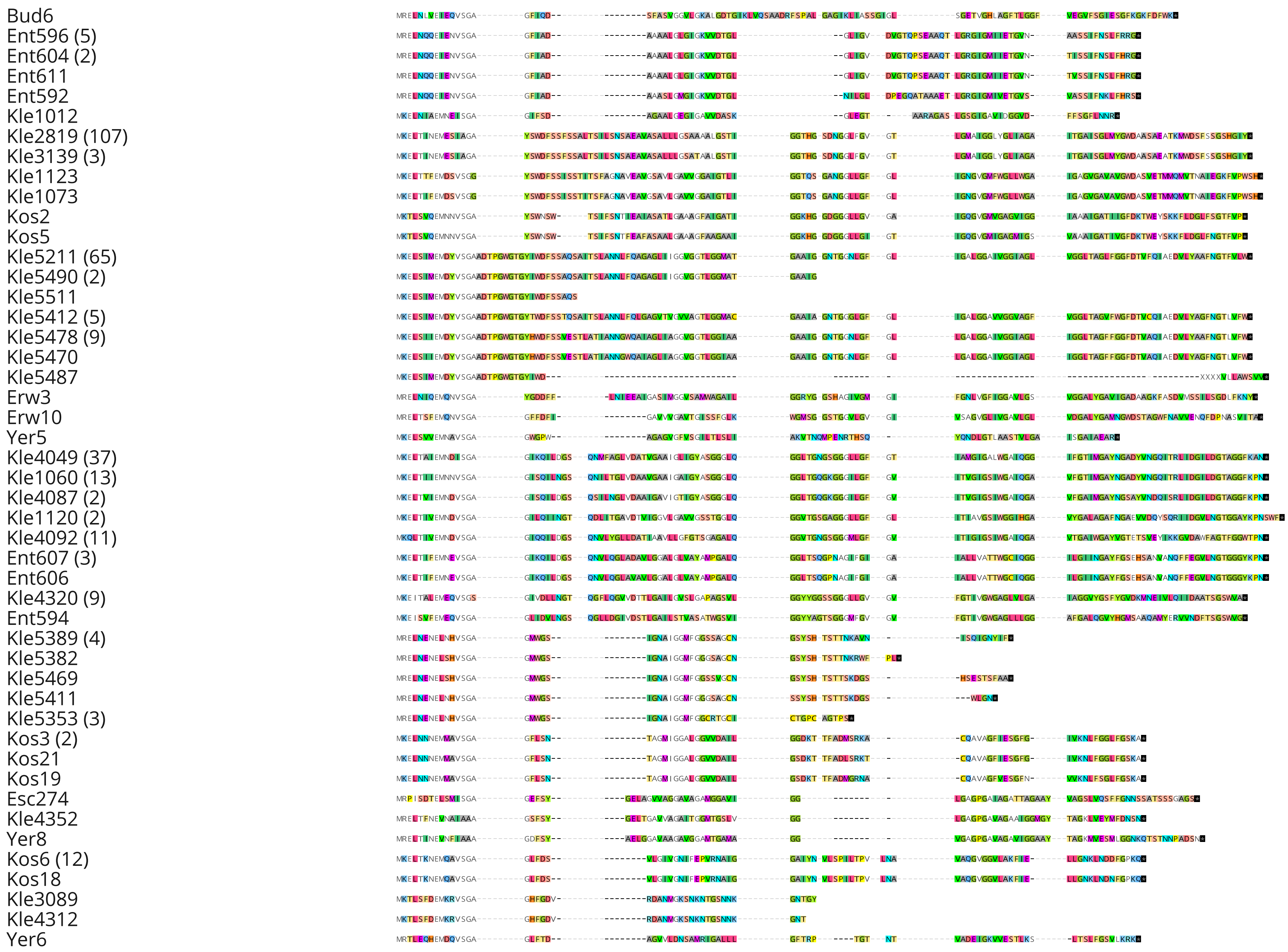
Bud6
Ent596 (5)
Ent604 (2)
Ent611
Ent592
Kle1012
Kle2819 (107)
Kle3139 (3)
Kle1123
Kle1073
Kos2
Kos5
Kle5211 (65)
Kle5490 (2)
Kle5511
Kle5412 (5)
Kle5478 (9)
Kle5470
Kle5487
Erw3
Erw10
Yer5
Kle4049 (37)
Kle1060 (13)
Kle4087 (2)
Kle1120 (2)
Kle4092 (11)
Ent607 (3)
Ent606
Kle4320 (9)
Ent594
Kle5389 (4)
Kle5382
Kle5469
Kle5411
Kle5353 (3)
Kos3 (2)
Kos21
Kos19
Esc274
Kle4352
Yer8
Kos6 (12)
Kos18
Kle3089
Kle4312
Yer6

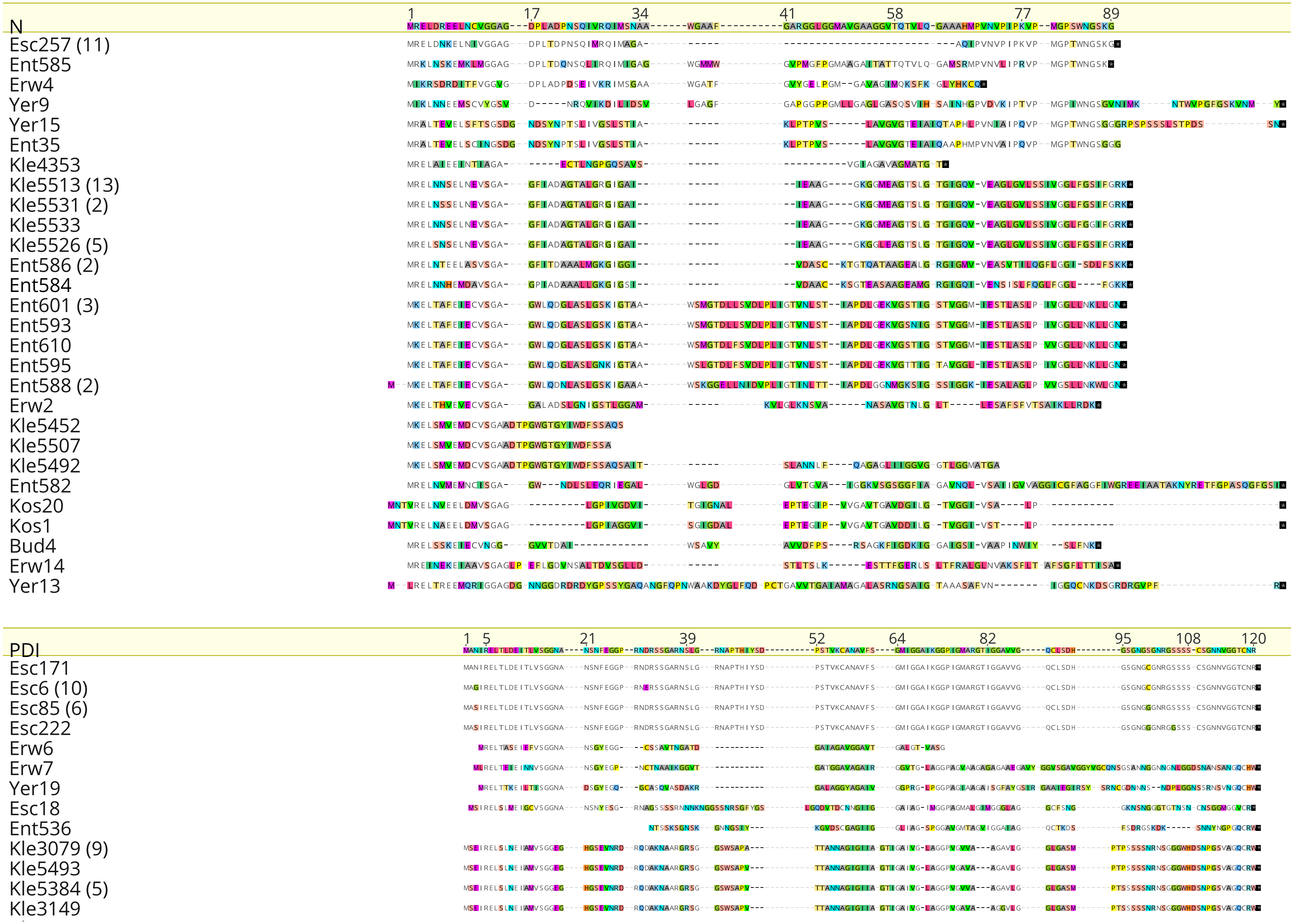
N
1
17
34
41
58
77
89
Esc257 (11)
Ent585
Erw4
Yer9
Yer15
Ent35
Kle4353
Kle5513 (13)
Kle5531 (2)
Kle5533
Kle5526 (5)
Ent586 (2)
Ent584
Ent601 (3)
Ent593
Ent610
Ent595
Ent588 (2)
Erw2
Kle5452
Kle5507
Kle5492
Ent582
Kos20
Kos1
Bud4
Erw14
Yer13
PDI
1
5
21
39
52
64
82
95
108
120
Esc171
Esc6 (10)
Esc85 (6)
Esc222
Erw6
Erw7
Yer19
Esc18
Ent536
Kle3079 (9)
Kle5493
Kle5384 (5)
Kle3149

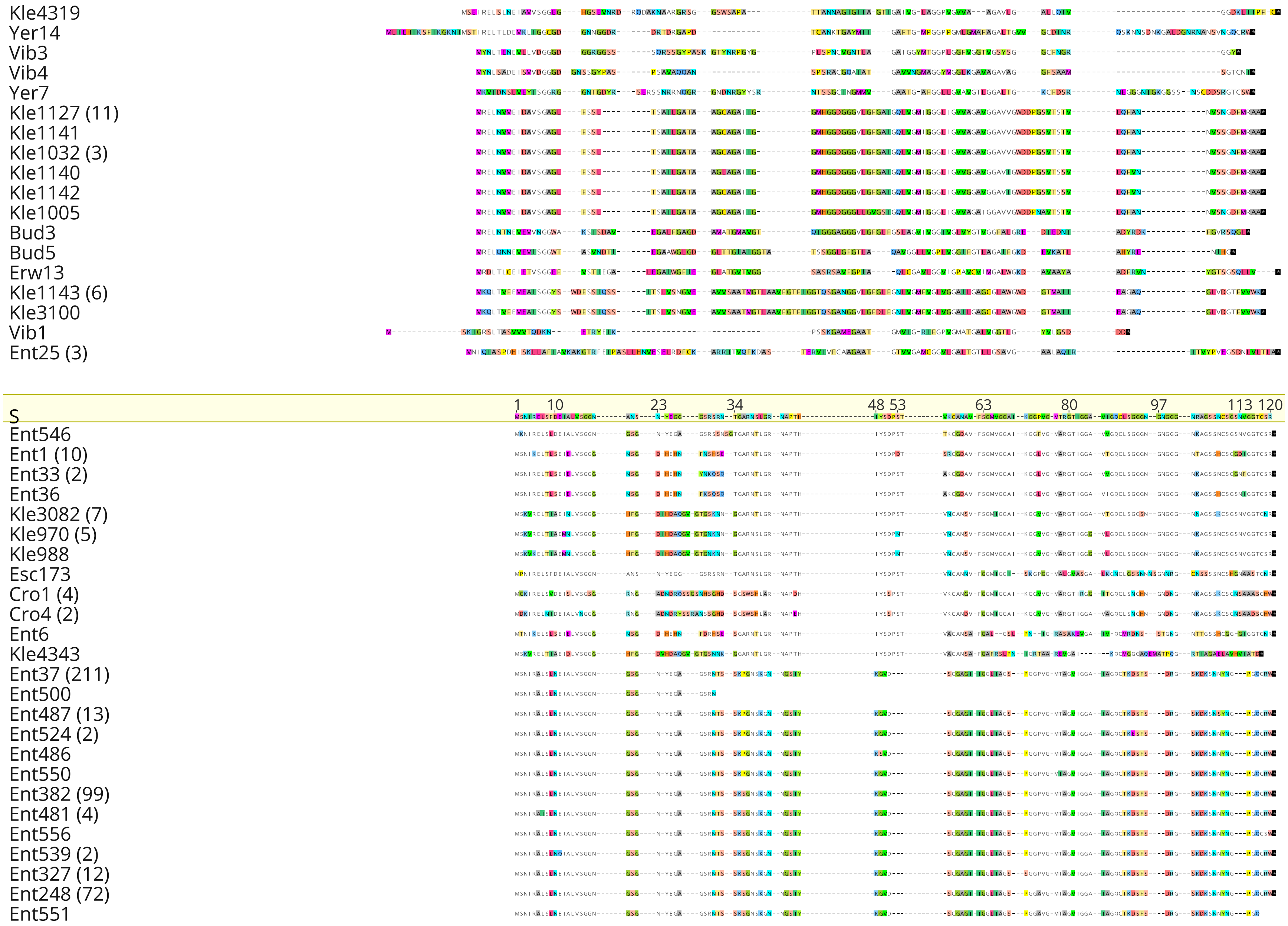

Kle4319
Yer14
Vib3
Vib4
Yer7
Kle1127 (11)
Kle1141
Kle1032 (3)
Kle1140
Kle1142
Kle1005
Bud3
Bud5
Erw13
Kle1143 (6)
Kle3100
Vib1
Ent25 (3)
S
1
10
23
34
48
53
63
80
97
113
120
Ent546
Ent1 (10)
Ent33 (2)
Ent36
Kle3082 (7)
Kle970 (5)
Kle988
Esc173
Cro1 (4)
Cro4 (2)
Ent6
Kle4343
Ent37 (211)
Ent500
Ent487 (13)
Ent524 (2)
Ent486
Ent550
Ent382 (99)
Ent481 (4)
Ent556
Ent539 (2)
Ent327 (12)
Ent248 (72)
Ent551

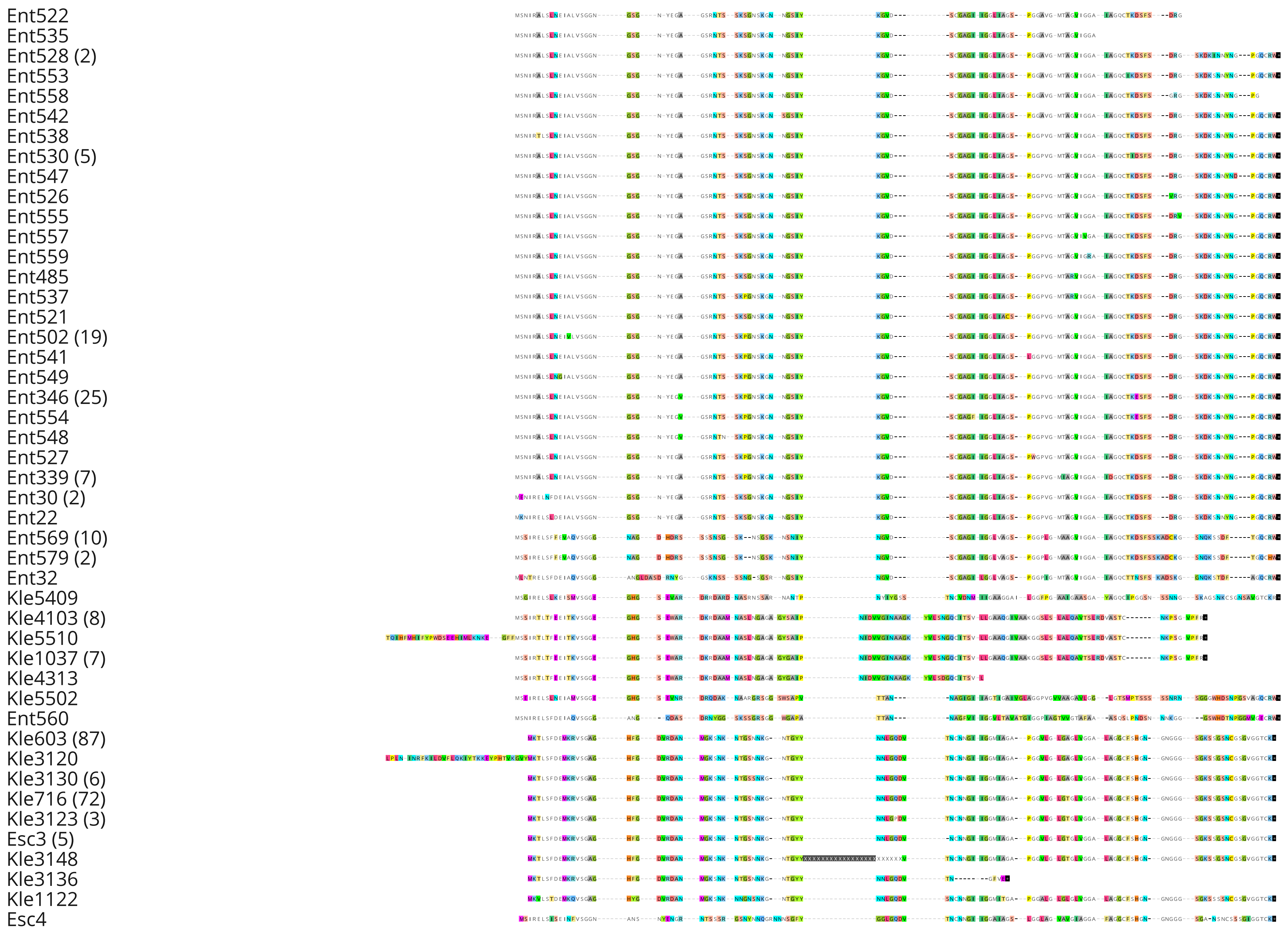
Ent522
Ent535
Ent528 (2)
Ent553
Ent558
Ent542
Ent538
Ent530 (5)
Ent547
Ent526
Ent555
Ent557
Ent559
Ent485
Ent537
Ent521
Ent502 (19)
Ent541
Ent549
Ent346 (25)
Ent554
Ent548
Ent527
Ent339 (7)
Ent30 (2)
Ent22
Ent569 (10)
Ent579 (2)
Ent32
Kle5409
Kle4103 (8)
Kle5510
Kle1037 (7)
Kle4313
Kle5502
Ent560
Kle603 (87)
Kle3120
Kle3130 (6)
Kle716 (72)
Kle3123 (3)
Esc3 (5)
Kle3148
Kle3136
Kle1122
Esc4

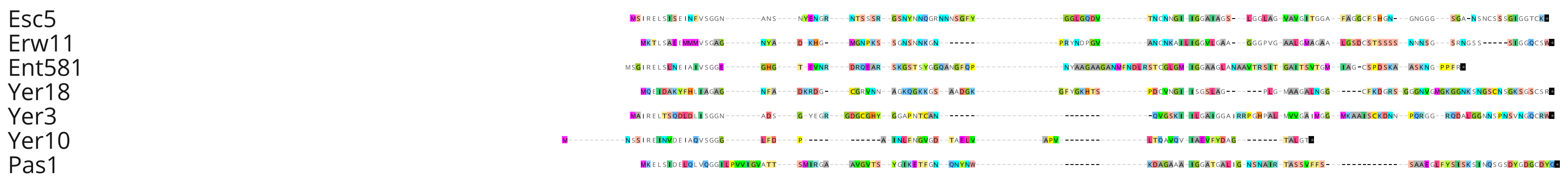

Class IIb Novel Microcin Hits

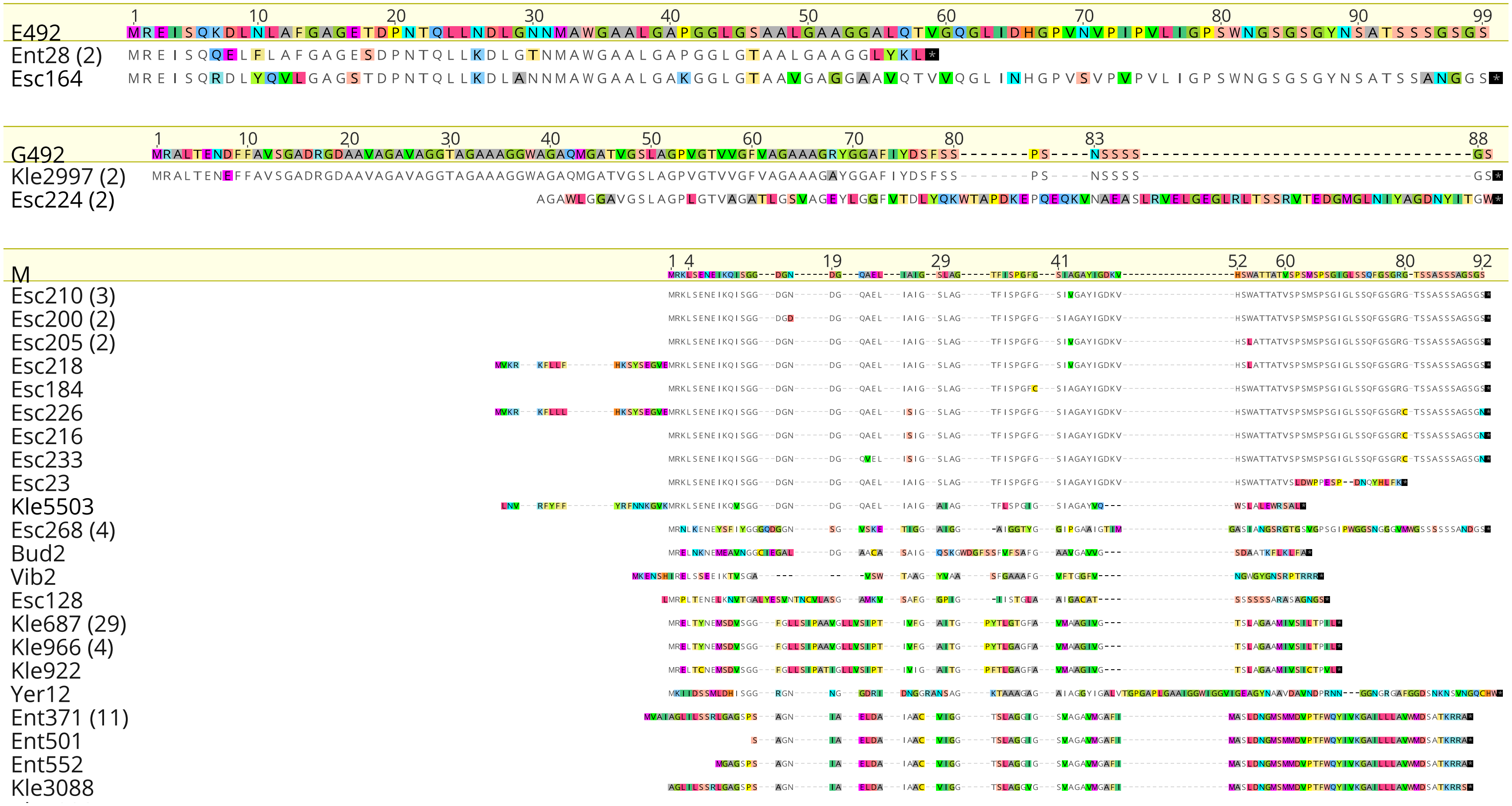

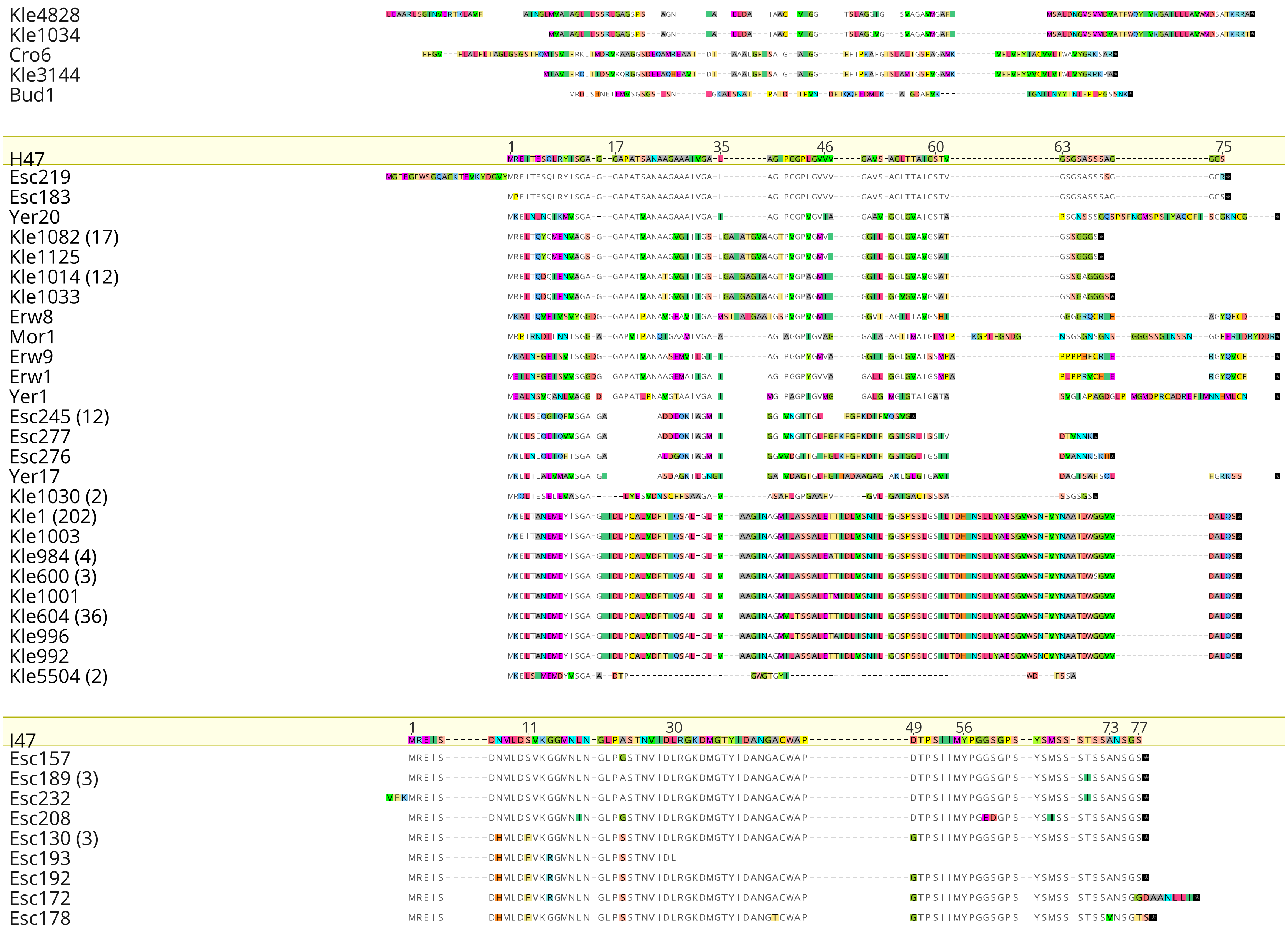
Kle4828
Kle1034
Cro6
Kle3144
Bud1
H47
1
17
35
46
60
63
75
Esc219
Esc183
Yer20
Kle1082 (17)
Kle1125
Kle1014 (12)
Kle1033
Erw8
Mor1
Erw9
Erw1
Yer1
Esc245 (12)
Esc277
Esc276
Yer17
Kle1030 (2)
Kle1 (202)
Kle1003
Kle984 (4)
Kle600 (3)
Kle1001
Kle604 (36)
Kle996
Kle992
Kle5504 (2)
I47
1
11
30
49
56
73
77
Esc157
Esc189 (3)
Esc232
Esc208
Esc130 (3)
Esc193
Esc192
Esc172
Esc178

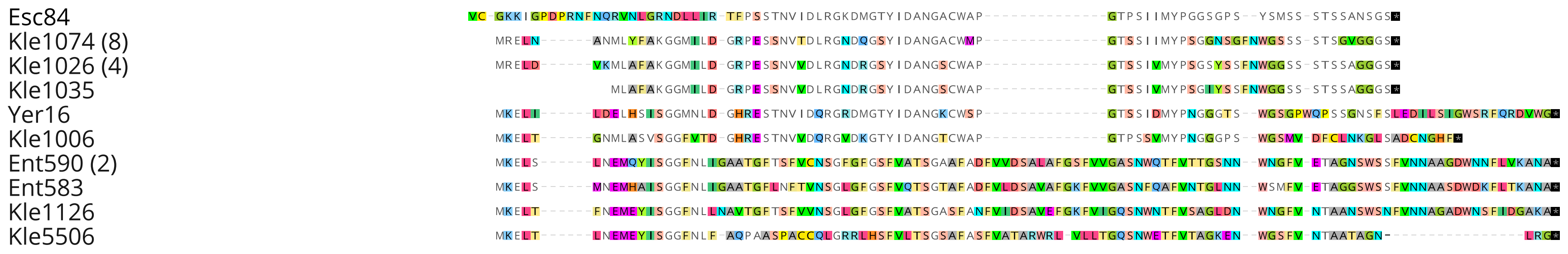
Esc84
Kle1074 (8)
Kle1026 (4)
Kle1035
Yer16
Kle1006
Ent590 (2)
Ent583
Kle1126
Kle5506

Supplemental File 2C. Multiple sequence alignment of unique hits to microcins PDI and S identified by *cinful* among *Enterobacteriaceae* genomes. Only hits which occurred more than once (n=39) were included. Hits are identified by the *Enterobacteriaceae* clade from which they originated. Numbers in parentheses indicate the number of occurrences of that particular sequence. The alignment also contains sequences from the 10 verified microcins for reference and from two Gram-positive bacteriocins (pediocin and piscicolin) as the outgroup. Agreements to the majority consensus sequence are highlighted.

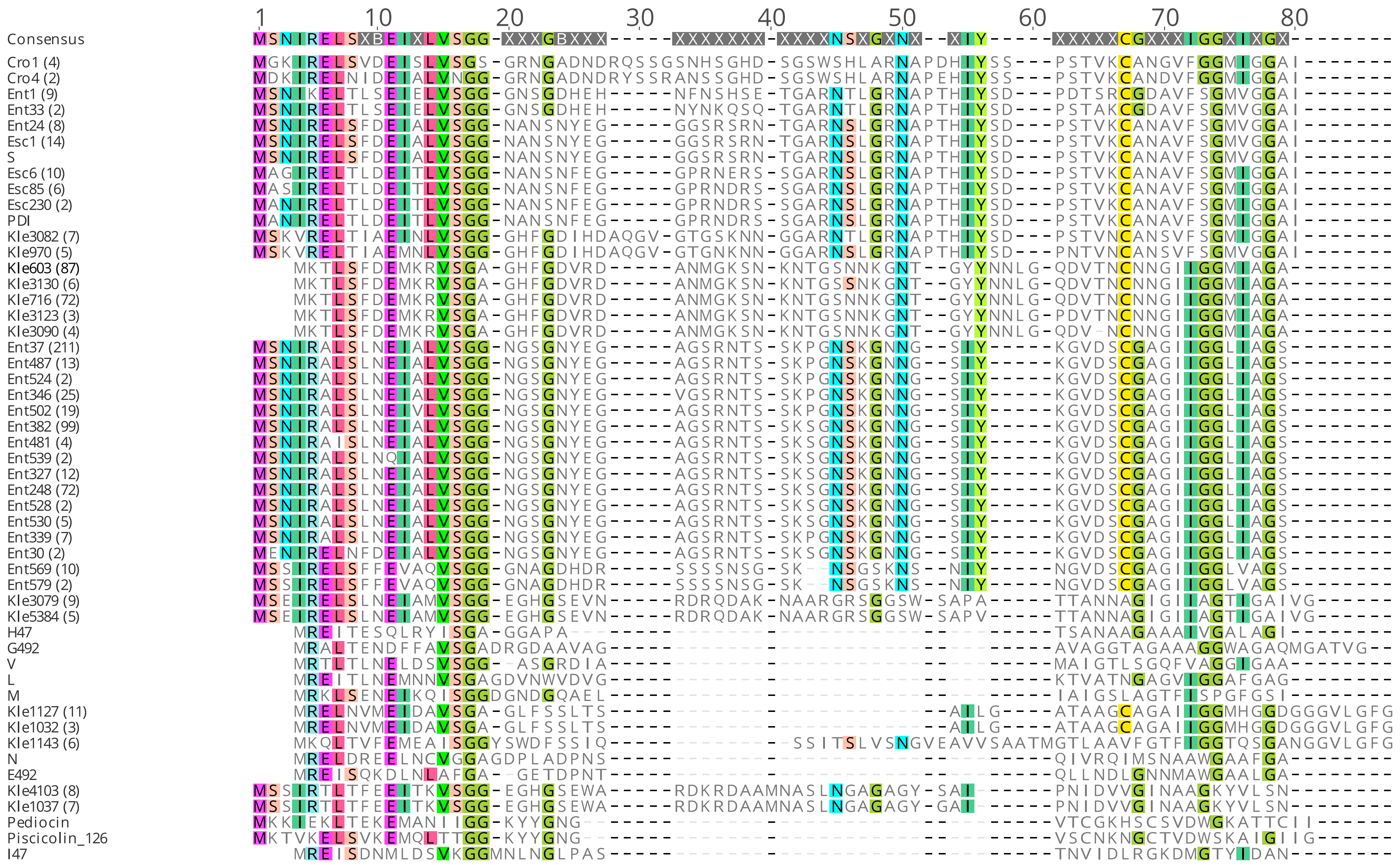

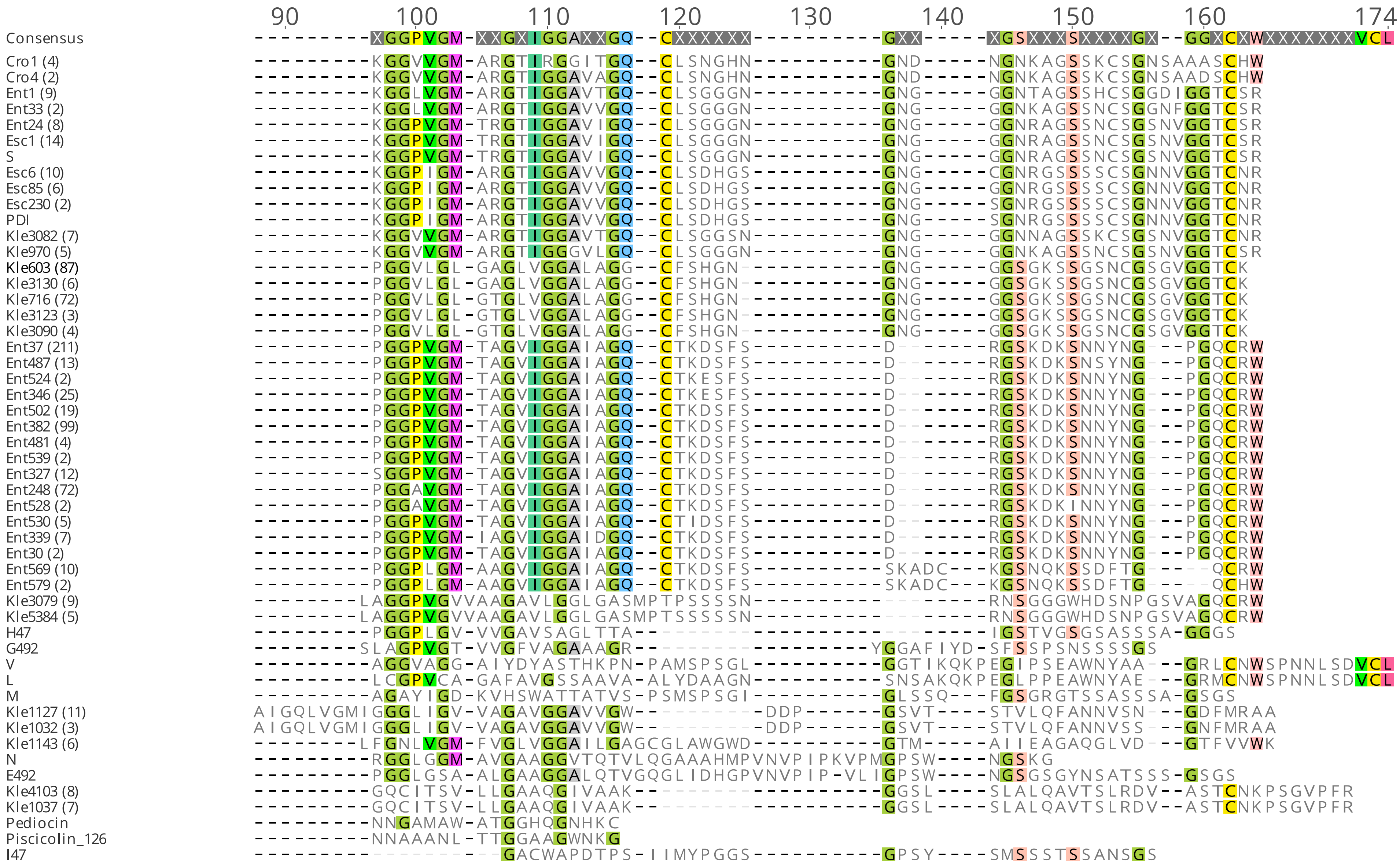

90
100
110
120
130
140
150
160
174
Consensus
Cro1 (4)
Cro4 (2)
Ent1 (9)
Ent33 (2)
Ent24 (8)
Esc1 (14)
S
Esc6 (10)
Esc85 (6)
Esc230 (2)
PDI
Kle3082 (7)
Kle970 (5)
Kle603 (87)
Kle3130 (6)
Kle716 (72)
Kle3123 (3)
Kle3090 (4)
Ent37 (211)
Ent487 (13)
Ent524 (2)
Ent346 (25)
Ent502 (19)
Ent382 (99)
Ent481 (4)
Ent539 (2)
Ent327 (12)
Ent248 (72)
Ent528 (2)
Ent530 (5)
Ent339 (7)
Ent30 (2)
Ent569 (10)
Ent579 (2)
Kle3079 (9)
Kle5384 (5)
H47
G492
V
L
M
Kle1127 (11)
Kle1032 (3)
Kle1143 (6)
N
E492
Kle4103 (8)
Kle1037 (7)
Pediocin
Piscicolin_126
I47

Supplemental File 2D. Multiple sequence alignment of all unique microcin hits identified by cinful among Enterobacterales, *Vibrionaceae*, and *Pasteurellaceae* genomes. Hits to both novel microcins (n=372) and verified microcins (n=10) are included. Hits are identified by taxon from which they originated: by major clade for hits from the *Enterobacteriaceae* or by family for hits outside of the *Enterobacteriaceae*. Numbers in parentheses indicate the number of occurrences of that particular sequence.The alignment also contains sequences from the 10 verified microcins for reference and from two Gram-positive bacteriocins (pediocin and piscicolin) as the outgroup. Agreements to the majority consensus sequenceare highlighted.

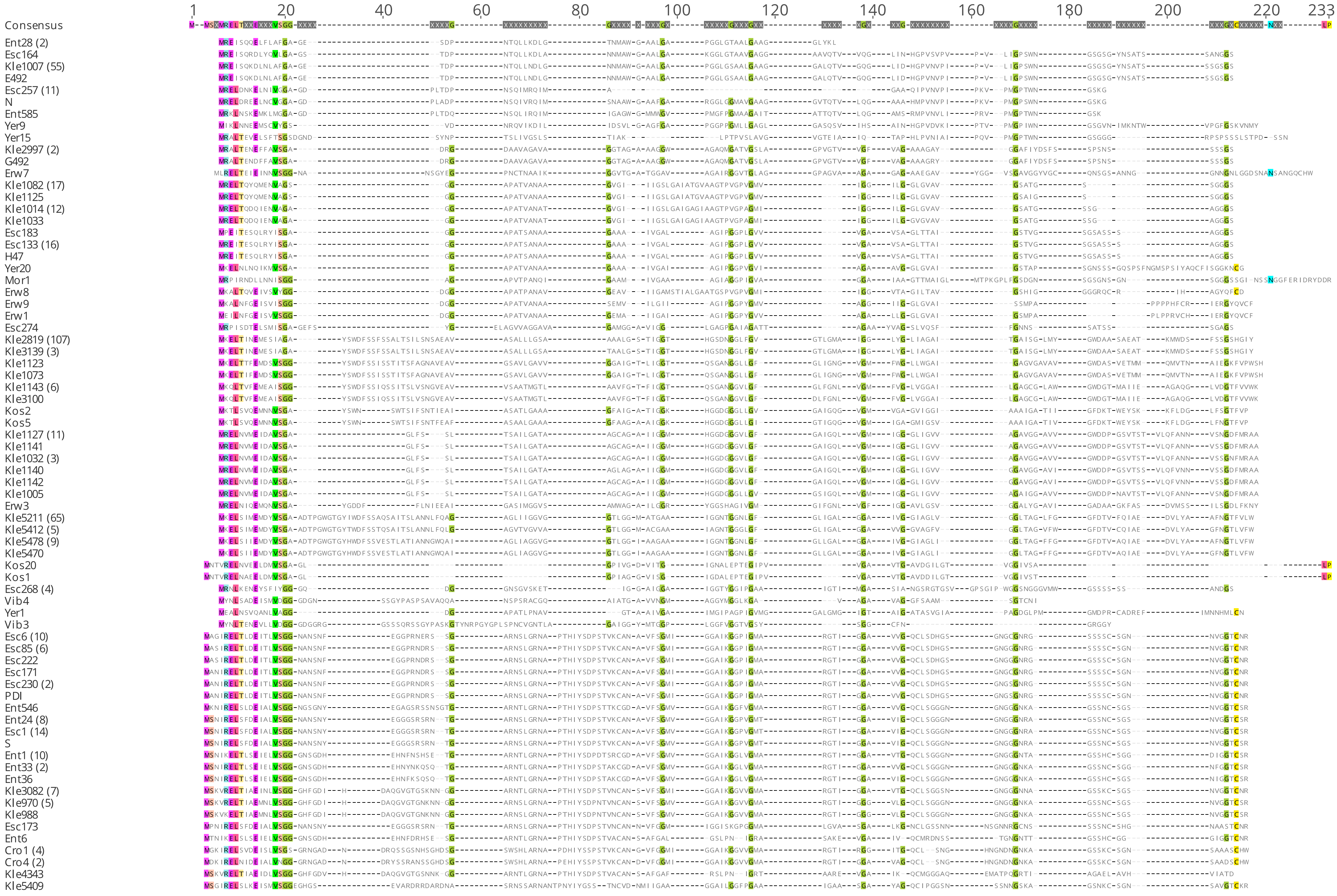

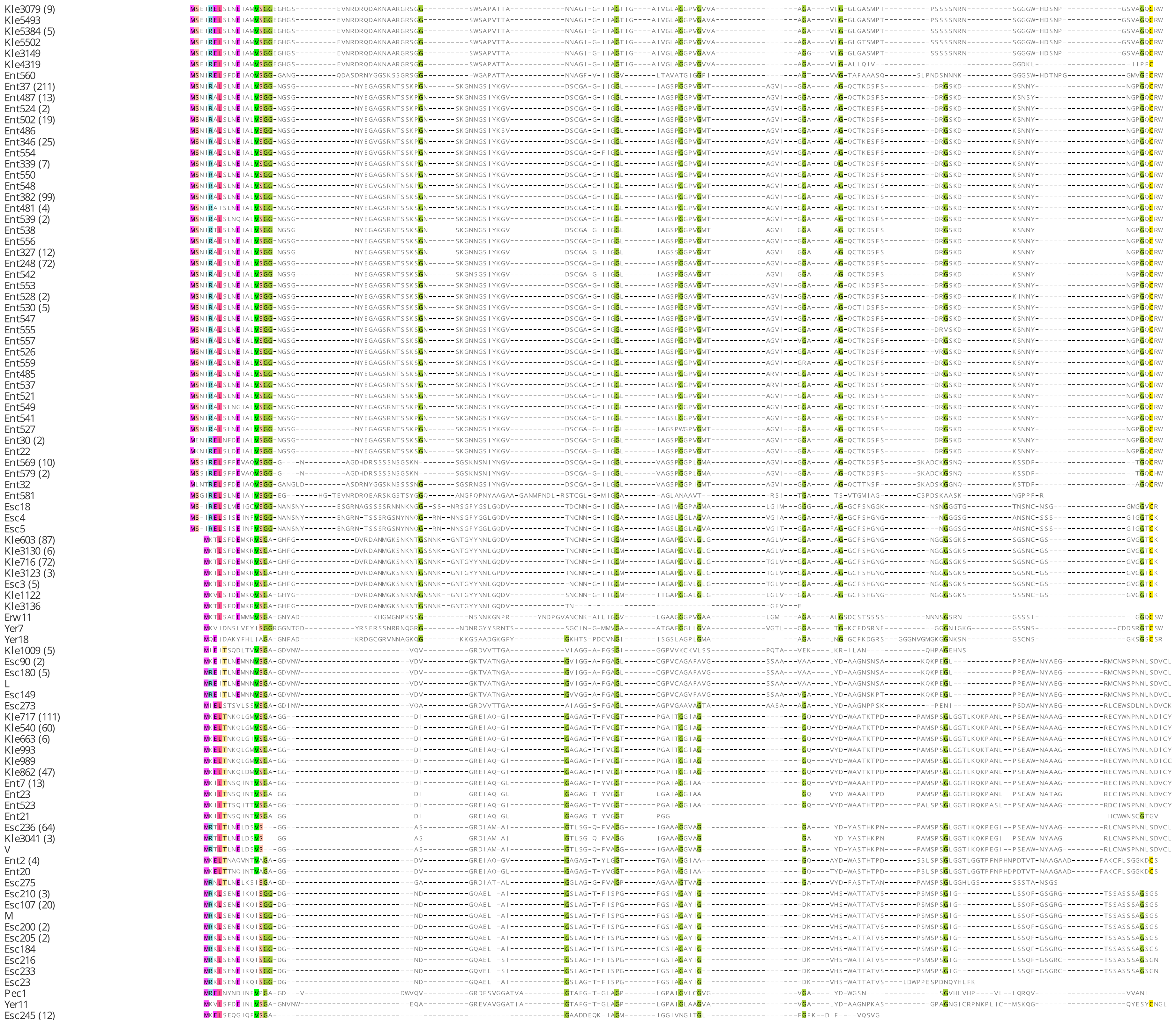

Kle3079 (9)
Kle5493
Kle5384 (5)
Kle5502
Kle3149
Kle4319
Ent560
Ent37 (211)
Ent487 (13)
Ent524 (2)
Ent502 (19)
Ent486
Ent346 (25)
Ent554
Ent339 (7)
Ent550
Ent548
Ent382 (99)
Ent481 (4)
Ent539 (2)
Ent538
Ent556
Ent327 (12)
Ent248 (72)
Ent542
Ent553
Ent528 (2)
Ent530 (5)
Ent547
Ent555
Ent557
Ent526
Ent559
Ent485
Ent537
Ent521
Ent549
Ent541
Ent527
Ent30 (2)
Ent22
Ent569 (10)
Ent579 (2)
Ent32
Ent581
Esc18
Esc4
Esc5
Kle603 (87)
Kle3130 (6)
Kle716 (72)
Kle3123 (3)
Esc3 (5)
Kle1122
Kle3136
Erw11
Yer7
Yer18
Kle1009 (5)
Esc90 (2)
Esc180 (5)
L
Esc149
Esc273
Kle717 (111)
Kle540 (60)
Kle663 (6)
Kle993
Kle989
Kle862 (47)
Ent7 (13)
Ent23
Ent523
Ent21
Esc236 (64)
Kle3041 (3)
V
Ent2 (4)
Ent20
Esc275
Esc210 (3)
Esc107 (20)
M
Esc200 (2)
Esc205 (2)
Esc184
Esc216
Esc233
Esc23
Pec1
Yer11
Esc245 (12)

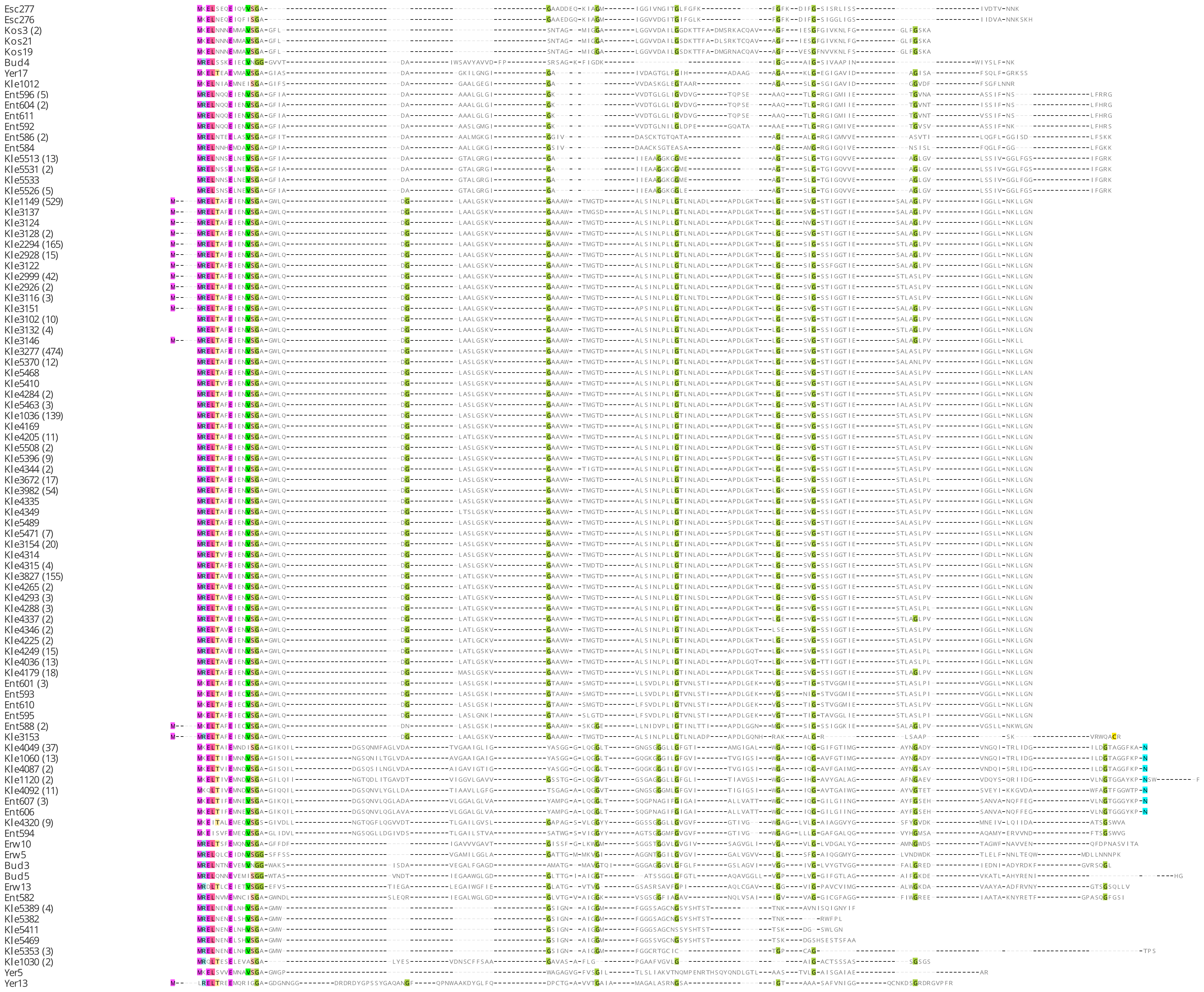

Esc277
Esc276
Kos3 (2)
Kos21
Kos19
Bud4
Yer17
Kle1012
Ent596 (5)
Ent604 (2)
Ent611
Ent592
Ent586 (2)
Ent584
Kle5513 (13)
Kle5531 (2)
Kle5533
Kle5526 (5)
Kle1149 (529)
Kle3137
Kle3124
Kle3128 (2)
Kle2294 (165)
Kle2928 (15)
Kle3122
Kle2999 (42)
Kle2926 (2)
Kle3116 (3)
Kle3151
Kle3102 (10)
Kle3132 (4)
Kle3146
Kle3277 (474)
Kle5370 (12)
Kle5468
Kle5410
Kle4284 (2)
Kle5463 (3)
Kle1036 (139)
Kle4169
Kle4205 (11)
Kle5508 (2)
Kle5396 (9)
Kle4344 (2)
Kle3672 (17)
Kle3982 (54)
Kle4335
Kle4349
Kle5489
Kle5471 (7)
Kle3154 (20)
Kle4314
Kle4315 (4)
Kle3827 (155)
Kle4265 (2)
Kle4293 (3)
Kle4288 (3)
Kle4337 (2)
Kle4346 (2)
Kle4225 (2)
Kle4249 (15)
Kle4036 (13)
Kle4179 (18)
Ent601 (3)
Ent593
Ent610
Ent595
Ent588 (2)
Kle3153
Kle4049 (37)
Kle1060 (13)
Kle4087 (2)
Kle1120 (2)
Kle4092 (11)
Ent607 (3)
Ent606
Kle4320 (9)
Ent594
Erw10
Erw5
Bud3
Bud5
Erw13
Ent582
Kle5389 (4)
Kle5382
Kle5411
Kle5469
Kle5353 (3)
Kle1030 (2)
Yer5
Yer13

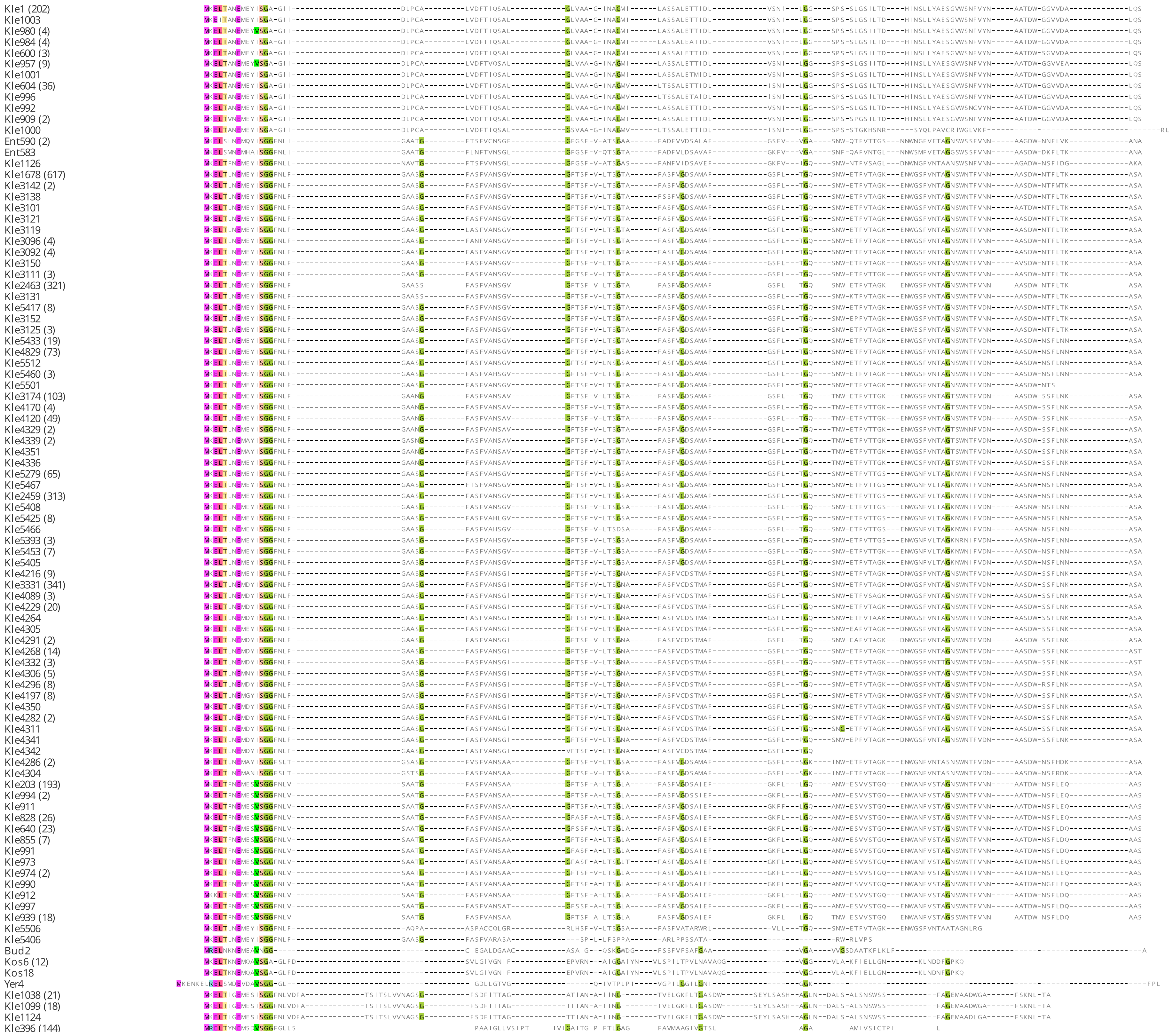

Kle1 (202)
Kle1003
Kle980 (4)
Kle984 (4)
Kle600 (3)
Kle957 (9)
Kle1001
Kle604 (36)
Kle996
Kle992
Kle909 (2)
Kle1000
Ent590 (2)
Ent583
Kle1126
Kle1678 (617)
Kle3142 (2)
Kle3138
Kle3101
Kle3121
Kle3119
Kle3096 (4)
Kle3092 (4)
Kle3150
Kle3111 (3)
Kle2463 (321)
Kle3131
Kle5417 (8)
Kle3152
Kle3125 (3)
Kle5433 (19)
Kle4829 (73)
Kle5512
Kle5460 (3)
Kle5501
Kle3174 (103)
Kle4170 (4)
Kle4120 (49)
Kle4329 (2)
Kle4339 (2)
Kle4351
Kle4336
Kle5279 (65)
Kle5467
Kle2459 (313)
Kle5408
Kle5425 (8)
Kle5466
Kle5393 (3)
Kle5453 (7)
Kle5405
Kle4216 (9)
Kle3331 (341)
Kle4089 (3)
Kle4229 (20)
Kle4264
Kle4305
Kle4291 (2)
Kle4268 (14)
Kle4332 (3)
Kle4306 (5)
Kle4296 (8)
Kle4197 (8)
Kle4350
Kle4282 (2)
Kle4311
Kle4341
Kle4342
Kle4286 (2)
Kle4304
Kle203 (193)
Kle994 (2)
Kle911
Kle828 (26)
Kle640 (23)
Kle855 (7)
Kle991
Kle973
Kle974 (2)
Kle990
Kle912
Kle997
Kle939 (18)
Kle5506
Kle5406
Bud2
Kos6 (12)
Kos18
Yer4
Kle1038 (21)
Kle1099 (18)
Kle1124
Kle396 (144)

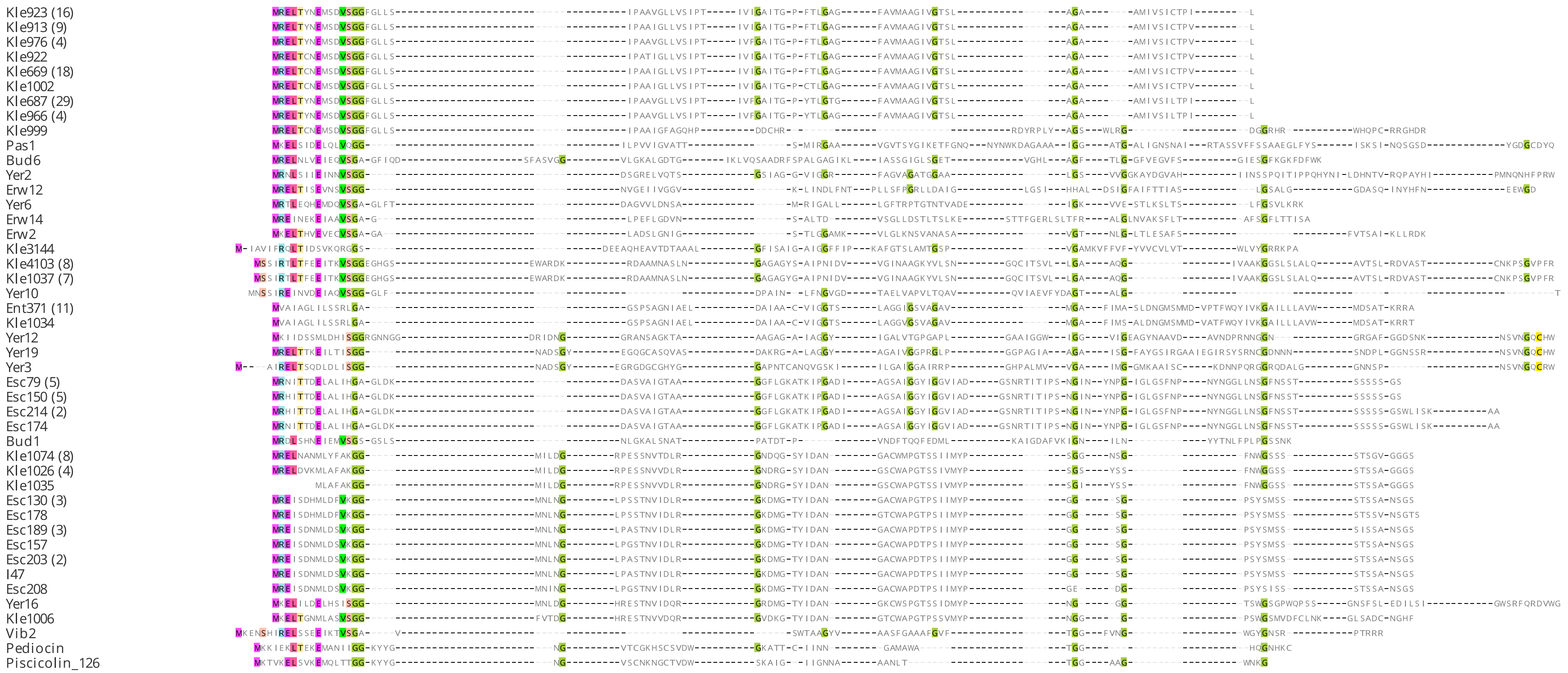
Kle923 (16)
Kle913 (9)
Kle976 (4)
Kle922
Kle669 (18)
Kle1002
Kle687 (29)
Kle966 (4)
Kle999
Pas1
Bud6
Yer2
Erw12
Yer6
Erw14
Erw2
Kle3144
Kle4103 (8)
Kle1037 (7)
Yer10
Ent371 (11)
Kle1034
Yer12
Yer19
Yer3
Esc79 (5)
Esc150 (5)
Esc214 (2)
Esc174
Bud1
Kle1074 (8)
Kle1026 (4)
Kle1035
Esc130 (3)
Esc178
Esc189 (3)
Esc157
Esc203 (2)
I47
Esc208
Yer16
Kle1006
Vib2
Pediocin
Piscicolin_126
